# Construction and analysis of competing endogenous RNA network and patterns of immune infiltration in abdominal aortic aneurysm

**DOI:** 10.3389/fcvm.2022.955838

**Published:** 2022-08-04

**Authors:** Liang Chen, Shuangshuang Wang, Zheyu Wang, Yuting Liu, Yi Xu, Shuofei Yang, Guanhua Xue

**Affiliations:** ^1^Department of Vascular Surgery, Renji Hospital, School of Medicine, Shanghai Jiaotong University, Shanghai, China; ^2^Songyuan Central Hospital, Songyuan Children's Hospital, Songyuan, China

**Keywords:** abdominal aortic aneurysm, circular RNA, competing endogenous RNA network, immune cell infiltration, bioinformatic analysis

## Abstract

**Background:**

Various studies have highlighted the role of circular RNAs (circRNAs) as critical molecular regulators in cardiovascular diseases, but its role in abdominal aortic aneurysm (AAA) is unclear. This study explores the potential molecular mechanisms of AAA based on the circRNA-microRNA (miRNA)-mRNA competing endogenous RNA (ceRNA) network and immune cell infiltration patterns.

**Methods:**

The expression profiles of circRNAs (GSE144431) and mRNAs (GSE57691 and GSE47472) were obtained from the Gene Expression Omnibus (GEO). Then, the differentially expressed circRNAs (DEcircRNAs) and mRNAs (DEmRNAs) between AAA patients and healthy control samples, and the target miRNAs of these DEmRNAs and DEcircRNAs were identified. Based on the miRNA-DEmRNAs and miRNA-DEcircRNAs pairs, the ceRNA network was constructed. Furthermore, the proportion of the 22 immune cell types in AAA patients was assessed using cell type identification by estimating relative subsets of RNA transcripts (CIBERSORT) algorithm. The expressions of key genes and immune cell infiltration were validated using clinical specimens.

**Results:**

A total of 214 DEmRNAs were identified in the GSE57691 and GSE47472 datasets, and 517 DEcircRNAs were identified in the GSE144431 dataset. The ceRNA network included 19 circRNAs, 36 mRNAs, and 68 miRNAs. Two key genes, *PPARG* and *FOXO1*, were identified among the hub genes of the established protein-protein interaction between mRNAs in the ceRNA network. Moreover, seven types of immune cells were differentially expressed between AAA patients and healthy control samples. Hub genes in ceRNA, such as *FOXO1, HSPA8*, and *RAB5C*, positively correlated with resting CD4 memory T cells or M1 macrophages, or both.

**Conclusion:**

In conclusion, a ceRNA interaction axis was constructed. The composition of infiltrating immune cells was analyzed in the abdominal aorta of AAA patients and healthy control samples. This may help identify potential therapeutic targets for AAA.

## Introduction

Abdominal aortic aneurysm (AAA) is a progressive vascular accompanies the risk of rupturing the dilated aortic segment and is potentially lethal (1). However, the mechanism of AAA progression is unclear; hence it is essential to explore the underlying molecular causes of AAA that will help improve the diagnosis and treatment of AAA patients (2). Recent studies have shown the role of circular RNAs (circRNAs) in the pathogenesis of cardiovascular diseases (3, 4). circRNAs are a unique class of RNA molecules; they form a circular closed-loop structure by a covalent bond linkage by back-splicing linear RNA (5). It has been shown that circRNAs act as competitive endogenous RNA (ceRNA) and are involved in cardiovascular disease pathogenesis (6). In the ceRNA network, circRNAs compete with microRNAs (miRNAs) through miRNA response elements (MRE), thereby negatively regulating the mRNA expression of protein-coding genes (7). A unique circRNA-miRNA-mRNA interaction could be a potential mechanism for the development and progression of AAA.

The main pathological features of AAA include smooth muscle cell (SMC) dysfunction, inflammation, immune cell infiltration, and extracellular matrix remodeling (8). Studies have shown an association between circ-FNDC3B and angiotensin II (Ang II) induced SMC dysfunction, suggesting that circ-FNDC3B/miR-143-3p/ADAM10 axis may regulate AAA pathogenesis (9). Further investigation of circ-Sirt1/miR-132/212/SIRT1 in SMC phenotypic switching provided another perspective on the pathogenesis of AAA (10). Recently, Ma et al. showed the involvement of hsa_circ_0087352 in promoting the inflammatory response of macrophages in AAA. The target miRNAs and mRNAs were identified, and a ceRNA network of hsa_circ_0087352/ hsa-miR-149-5p/ IL-6 in AAA was constructed (11). Results suggest hsa_circ_0087352 promotes IL-6 transcription and secretion of inflammatory cytokine via endogenous hsa-miR-149-5p in macrophages thereby hsa_circ_0087352 could be potentially used in AAA therapeutics. These findings suggest that circRNA may have different targets and functions in other cells and tissues, and the circRNA-miRNA-mRNA network could play a significant role in AAA pathogenesis. Studies have proven the involvement of the infiltrating immune cells, such as neutrophils, macrophages, and T cells, in the occurrence and development of AAA (12). AAA is characterized by the infiltration of immune cells, suggesting that the immune system plays a critical role in AAA progression (13, 14). However, the ceRNA network and its association with infiltrating immune cells in AAA have not been thoroughly elucidated (15, 16).

This study aims to explore a novel circRNA-miRNA-mRNA ceRNA axis in AAA by analyzing microarray datasets from publicly available databases. Various patterns of immune cell infiltration in AAA were studied using the “cell type identification by estimating relative subsets of RNA transcripts (CIBERSORT)” algorithm. The co-expression patterns of immune cells and hub genes of the ceRNA network were also identified. Moreover, the target mRNAs of DEcircRNAs and immune infiltration were validated in healthy control samples and AAA patients. This study sheds light on the potential role of ceRNA in the pathogenesis of AAA and its underlying immune infiltration signature.

## Materials and methods

### Data collection and differential expression analysis

The microarray data of AAA were retrieved from the National Center for Biotechnology Information Gene Expression Omnibus (NCBI_GEO) database (https://www.ncbi.nlm.nih.gov/geo/). The species type as “Homo sapiens.” was set as a filter and the results obtained included three datasets GSE47472, GSE57691, and GSE144431. The data of circRNA expression (GSE144431) was ncRNA profiling by array, and the data of the microarray platform by 074301 Arraystar Human ncRNA microarray V2 (platform: GPL21825), the mRNA expression datasets (GSE47472 and GSE57691) by expression profiling by array, and the data of the microarray platform by Illumina HumanHT-12 V4.0 expression BeadChip (platform: GPL10558). Each dataset includes data from patients with AAA and normal aorta (which will be referred to as the healthy control group). Series matrix files and expressive data were retrieved from the GEO database.

The differentially expressed mRNAs (DEmRNAs) and circRNAs (DEcircRNAs) between the AAA patients and the healthy control group were analyzed and compared using the “Linear Models for Microarray Data (limma)” R package. mRNAs and circRNAs were considered as DEmRNAs and DEcircRNAs if they met the criteria: |log2 fold change (FC)| > 1 and false discovery rate (FDR) adjusted *p*-value < 0.05. The differential analysis results were presented as volcano plots and heat maps, and related tabular information was derived.

### Functional enrichment and pathway analysis

Kyoto Encyclopedia of Genes and Genomes (KEGG) enrichment analysis and Gene Ontology (GO) were used to explore the biological functions of DEmRNAs in AAA. The KEGG enrichment analysis and GO analysis included biological processes (BP), molecular functions (MF), and cellular components (CC), and the analysis was performed using the “clusterProfiler” package of the R software. FDR adjusted *p*-value < 0.05 was considered statistically significant.

### Construction of the ceRNA network

The DEcircRNAs (GSE144431) and DEmRNAs (GSE57691 and GSE47472) between AAA patients and healthy control samples were identified, and the target miRNAs of the key DEmRNAs and DEcircRNAs were predicted. The DEmRNA-miRNA pairs were predicted using the target miRNA information from the databases like miRcode, miRDB, TargetScan, miRmap, and miRanda databases (17–21). The DEcircRNA-miRNA pairs were determined using the starBase database (http://starbase.sysu.edu.cn/index.php). DEcircRNA-miRNA pairs and DEmRNA-miRNA pairs were used to construct the ceRNA network and visualized using cytoHubba in Cytoscape 3.4.0 software.

### Protein-protein interaction (PPI) network analysis and hub genes identification

The Search Tool for the Retrieval of Interacting Genes (STRING; version 11.0) was used to explore the protein-protein interactions between mRNAs in the ceRNA network (22). The PPI network of the DEmRNAs with combined score >0.4 in STRING was considered as a functional link. The PPI network was visualized by Cytoscape 3.4.0. The cytoHubba plugin of the Cytoscape software calculates the dense relationship through the degree, betweenness centrality, and closeness centrality algorithms. The hub genes of the PPI network were confirmed by cytoHubba.

### Analysis of immune cell infiltration

The mRNA microarray dataset GSE57691 was analyzed to study the proportion of 22 infiltrating immune cells in the tissues between AAA patients and the healthy control samples using the CIBERSORT algorithm (23). The significantly different cell types (*p* < 0.05) between AAA patients and the healthy control samples were filtered out in the CIBERSORT analysis. Subsequently, the Wilcoxon rank-sum test was applied to assess differentially infiltrating immune cells between patients with AAA and controls, visualized by “heatmap” and “Violin plot” packages of the R software.

### Human studies

The study was approved by the Institutional Review Board of the Shanghai Jiaotong University School of Medicine, Renji Hospital (No. KY2021–168). All experiments were conducted in accordance with the Declaration of Helsinki. Written informed consent was obtained from all patients or their families before the collection of the biological specimens. The presence of an aortic aneurysm was confirmed prior to the echocardiography (ECG) or computed tomography (CT)/magnetic resonance imaging (MRI). The clinical diagnosis was established before surgery by expert clinicians. Healthy control aortic samples were obtained from organ donors.

### Immunofluorescence

The abdominal aortas were harvested and fixed using 4% paraformaldehyde (PFA), embedded in paraffin, and serial sections of five μm thickness were prepared on poly-l-lysine coated slides. The sections were dewaxed, rehydrated, and permeabilized using 0.1% Triton X-100. The sections were then blocked using 1% goat serum for 1 h at room temperature and further incubated with anti-rabbit CD68 antibody (1:200, ab213363, Abcam, MA, USA) and anti-rabbit CD86 antibody (1:200, ab239075, Abcam, USA) at 4°C overnight. The following day, slides containing sections were incubated with fluorescent-conjugated secondary antibodies diluted in blocking buffer for 1 h at room temperature and mounted using 4′,6-diamidino-2-phenylindole (DAPI, Vector, ZsBio, Beijing, China). Imaging was done using a confocal microscope (Leica-SP8, Wetzlar, Germany).

### Quantitative reverse transcription polymerase chain reaction (qRT-PCR)

Total RNA from tissue samples was isolated using TRIzol (15596018, Invitrogen, Carlsbad, CA) per the manufacturer's instructions. Complementary DNA was synthesized using 500 ng of total RNA using the Reverse Transcription Kit (EZBioscience, MN, USA) per the manufacturer's instructions. qRT-PCR was performed using SYBR Green qPCR Master Mix (EZBioscience, MN, USA). Relative gene expression was calculated using the 2^−ΔΔCT^ method. PCR was performed using three biological and technical replicates and normalized using the housekeeping gene glyceraldehyde 3-phosphate dehydrogenase. Primer sequences are listed in Supplementary Table S1.

### Statistical analysis

The statistical analysis was carried out using Statistical Package for Social Sciences (SPSS, Chicago, Illinois) version 22.0. The data are expressed as the mean ± standard deviation or percentage. The variables between the two groups were compared using Student's *t*-test. Spearman's rank correlation analysis was performed to study the correlation between the hub genes of the ceRNA network and differentially expressed infiltrating immune cells. *p* < 0.05 was accepted as statistically significant.

## Results

### Identification of the DEmRNAs and DEcircRNAs

**Figure 1:**
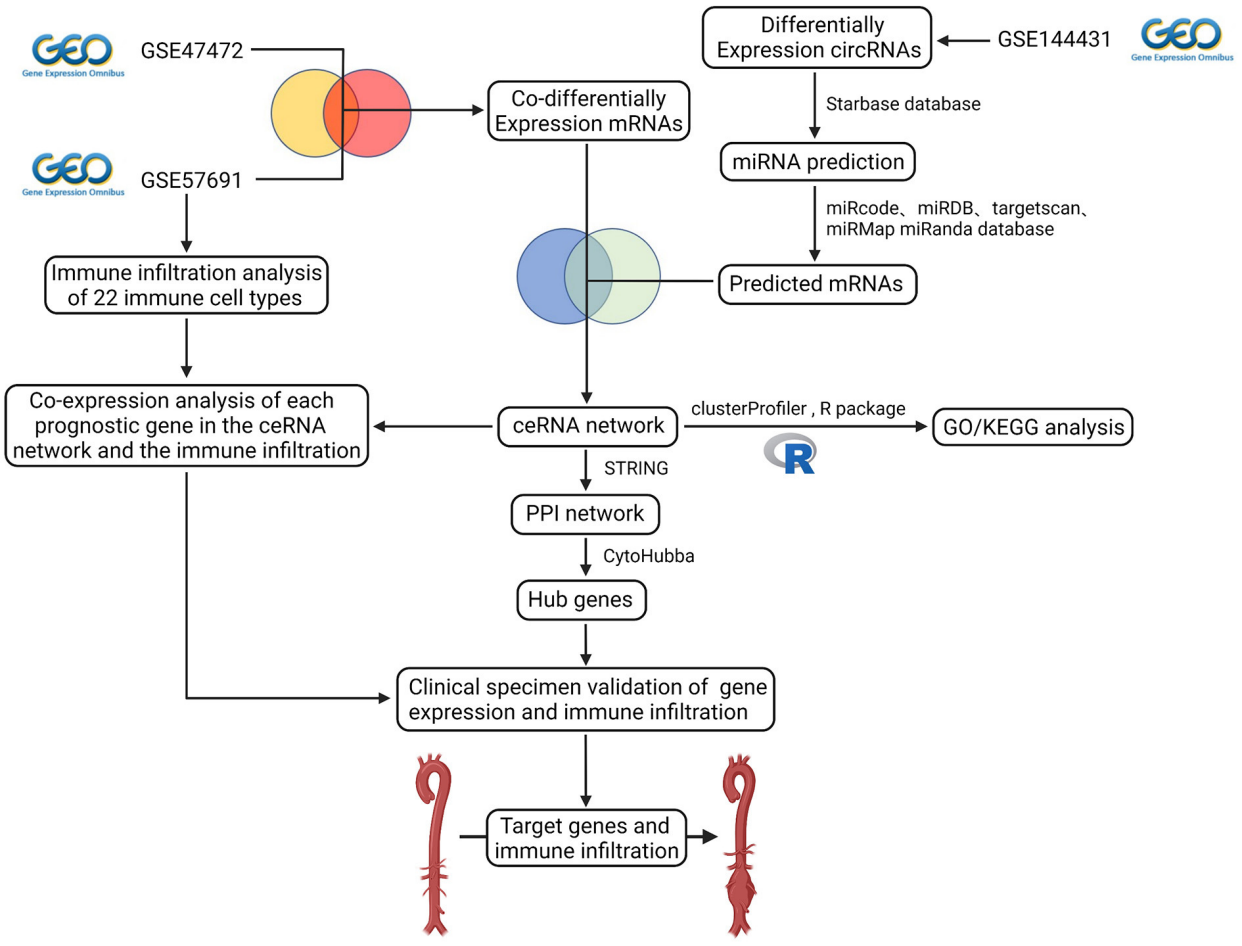
Flow diagram of the analytic process. The difference expression of three datasets (circRNA dataset and mRNA dataset) was analyzed. Construction of ceRNA network, protein-protein interaction network, functional enrichment analysis, co-expression analysis of essential genes and the immune infiltration, and validation of gene expression and immune infiltration.

**Figure 2:**
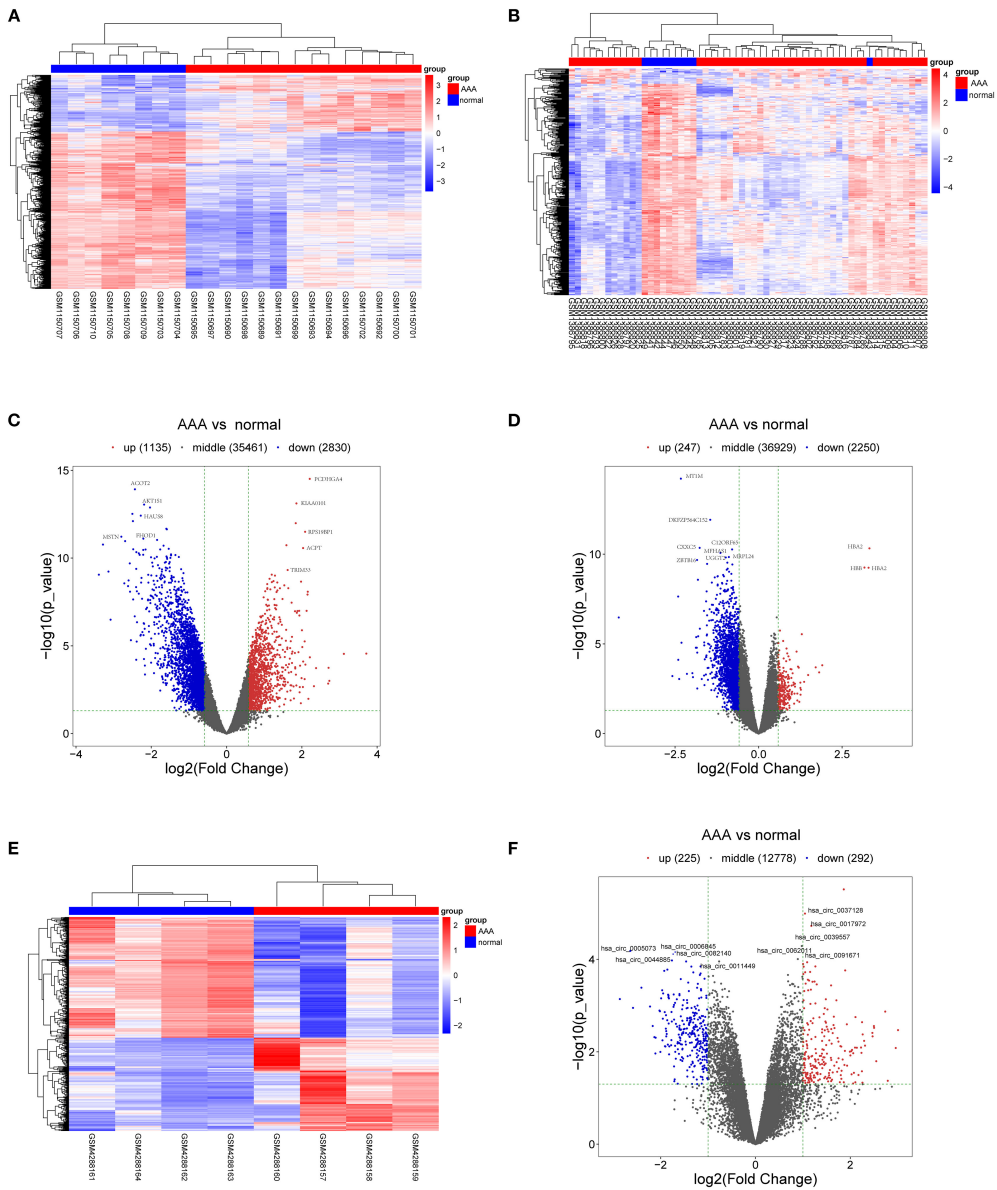
DEmRNAs and DEcircRNAs in aortic tissues between patients with AAA and normal controls. **(A)** The heat map of GSE47472, mRNAs. **(B)** The heat map of GSE57691, mRNAs. **(C)** The volcano plot of GSE47472, mRNAs. **(D)** The volcano plot of GSE57691, mRNAs. **(E)** The heat map of GSE144431, circRNAs. **(F)** The volcano plot of GSE144431, circRNAs. mRNAs, messenger RNAs; circRNAs, circular RNAs; DEmRNAs, differentially expressed messenger RNAs; DEcircRNAs, differentially expressed circular RNAs.

The schematic diagram in Figure 1 illustrates the course of this study. A total of 1,135 upregulated mRNA and 2830 downregulated mRNAs were identified in the GSE47472 dataset. The GSE57691 had 2497 DEmRNAs, of which 247 mRNAs were upregulated, and 2250 mRNAs were downregulated. DEmRNAs were visualized by hierarchical clustering heatmap analysis (Figures 2A,B) and volcano plots analysis (Figures 2C,D). In the GSE14431 dataset, 225 upregulated and 292 downregulated circRNAs were visualized by hierarchical clustering heat map analysis (Figure 2E) and volcano plot analysis (Figure 2F). The most significant differentially expressed DEmRNAs and DEcircRNAs were shown in Tables 1, 2.

**Table 1:** Top20 differentially expressed mRNA between normal tissue and AAAs.

	**GSE47472**			**GSE57691**	
**Gene symbol**	***P*-value**	**LogFC**	**Gene symbol**	***P*-value**	**LogFC**
DYNLL1	0.001007989	3.713704803	HBA2	1.60E-06	3.293491798
ANP32AP1	5.28308E-07	−3.390945819	HBB	1.60E-06	3.170975286
WDR82	3.87398E-08	−3.283955683	RNA28S5	0.012924033	−2.581261085
MSTN	1.81266E-08	−2.794857846	CTSZ	1.74E-05	−2.406532079
LEPR	1.0643E-06	−2.715442603	RPL10A	0.003290958	−2.405450673
ABI1	0.019371581	2.697968288	S100A4	0.016550251	−2.375031783
CARM1	2.61275E-08	−2.695557916	MT1M	2.45E-10	−2.325607304
BRINP3	0.000272617	−2.542055148	RPS17	0.010658672	−2.14452781
KAAG1	2.72774E-05	−2.439965976	RPL21	0.014103802	−1.963001154
ACOT2	2.33003E-10	−2.435730836	FOSB	0.005096434	1.907473194
FAM156A	0.001804728	−2.400505381	MT1X	0.001242665	−1.877340613
XPA	0.000312092	−2.387135524	PPP1R3C	0.000505801	−1.867351411
NAPSA	2.24906E-06	−2.381882488	UBB	0.001242665	−1.849839701
LSM2	4.99234E-05	−2.37779002	ZBTB16	9.16E-07	−1.84119325
ZNF575	7.6998E-08	−2.331880674	RPL26	0.037574325	−1.835326334
LARP7	0.024917017	2.31996564	SCRG1	0.00179909	−1.835280995
NR2F6	0.000175356	−2.286478187	IL6	0.009440574	1.815048907
CNGA3	4.85705E-06	−2.24473153	CXXC5	4.29E-07	−1.764643947
UBE3C	0.001423926	2.211969517	MAOA	0.000268881	−1.757136092
FHOD1	2.15387E-08	−2.211095781	SIK1	0.006202667	1.710707835

**Table 2:** Top20 differentially expressed circRNA between normal tissue and AAAs in GSE144431.

**circRNA**	***P*-value**	**LogFC**
hsa_circ_0001588	3.007621	0.111332
hsa_circ_0000517	2.957863	0.133612
hsa_circ_0092291	−2.8589	0.095681
hsa_circ_0092342	2.790772	0.231818
hsa_circ_0006156	2.736588	0.103484
hsa_circ_0005073	−2.63757	0.094948
hsa_circ_0090069	−2.5822	0.103484
hsa_circ_0000518	2.547719	0.159934
hsa_circ_0000524	2.501812	0.11042
hsa_circ_0007148	2.497819	0.109058
hsa_circ_0068655	2.470108	0.116696
hsa_circ_0008285	2.465435	0.112551
hsa_circ_0057691	−2.40486	0.094948
hsa_circ_0042268	2.291068	0.125054
hsa_circ_0000512	2.25612	0.242569
hsa_circ_0008410	2.245111	0.193686
hsa_circ_0009361	2.235248	0.141517
hsa_circ_0007249	2.226477	0.133612
hsa_circ_0003249	−2.22267	0.103484
hsa_circ_0014213	2.173152	0.20738

### GO and KEGG enrichment analysis of the DEmRNAs

**Figure 3:**
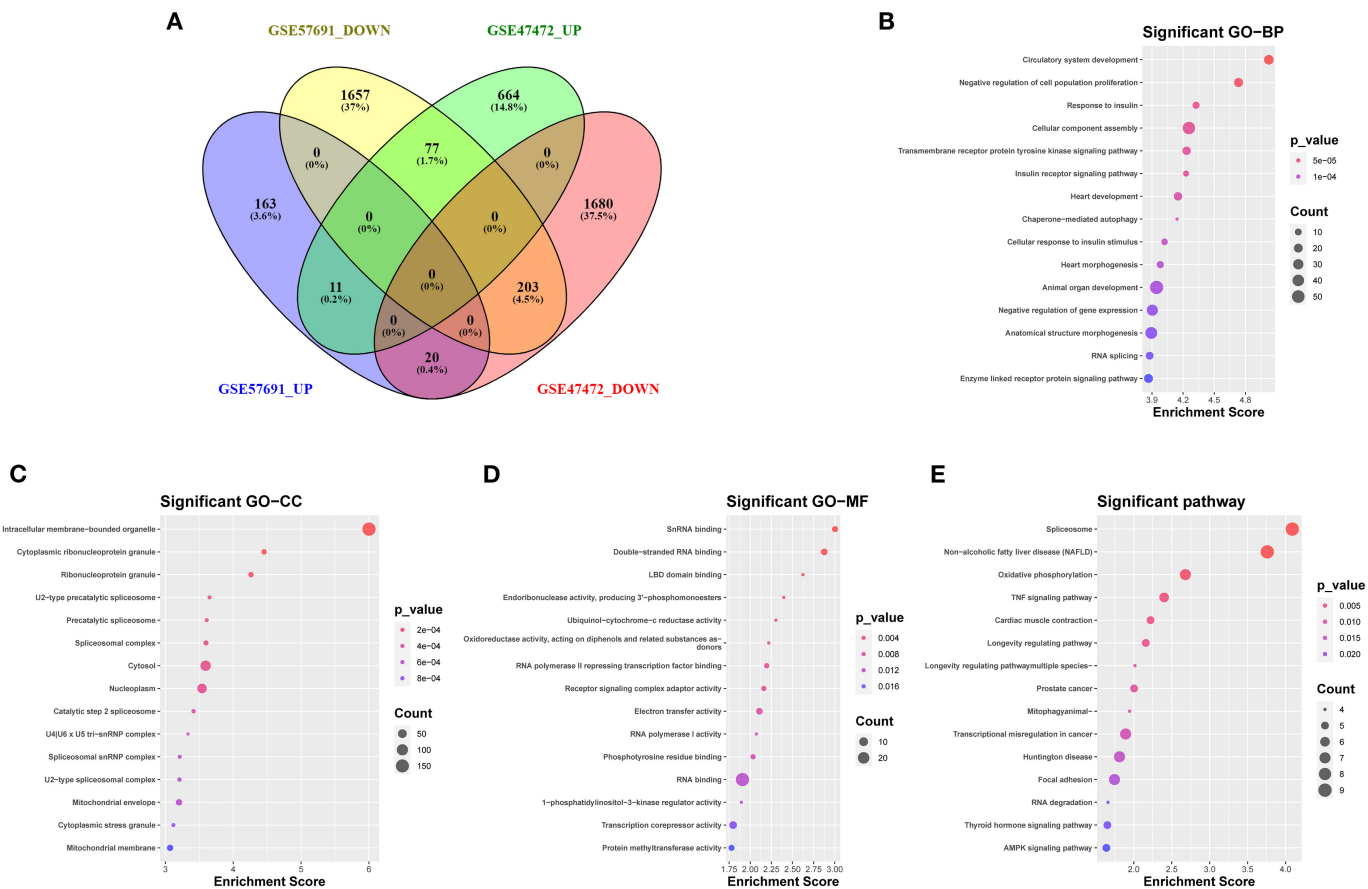
Venn diagram analysis and GO/KEGG analysis of 214 DEmRNAs coregulation modes in the two datasets **(A)** The intersection of downregulated and upregulated DEmRNAs (GSE57691 and GSE47472). **(B)** Biological process of 214 DEmRNAs by GO analysis. **(C)** Cellular component of 214 DEmRNAs by GO analysis. **(D)** Molecular function of 214 DEmRNAs by GO analysis. **(E)** KEGG enrichment analysis of DEmRNAs. GO, Gene Ontology; KEGG, Kyoto Encyclopedia of Genes and Genomes; DEmRNAs, differentially expressed messenger RNAs; BP, biological process; CC, cellular component; MF, molecular function.

The Venn diagram showed 214 overlapping DEmRNAs, of which 11 mRNAs were upregulated, and 203 mRNA were downregulated between GSE47472 and GSE57691 (Figure 3A). GO analysis of these DEmRNAs indicated that BP was significantly enriched in the circulatory system development, negative regulation of cell population proliferation, and response to insulin (Figure 3B; Supplementary Figure S1). The CC ontology cellular processes that were enriched were related to the intracellular membrane-bounded organelle, cytoplasmic ribonucleoprotein granule, and ribonucleoprotein granule (Figure 3C; Supplementary Figure S2). In the MF ontology, enriched processes included snRNA binding, double-stranded RNA binding, and ligand-binding domain (LBD) domain binding (Figure 3D; Supplementary Figure S3). Additional GO analysis was shown in (Supplementary Figure S4. Furthermore, KEGG pathway analysis revealed that these DEmRNAs are mainly involved in the spliceosome, non-alcoholic fatty liver disease (NAFLD), oxidative phosphorylation, and tumor necrosis factor (TNF) signaling pathway (Figure 3E; Supplementary Figure S5). GO and KEGG enrichment analyses are presented in Supplementary Tables S2, S3.

### Construction of the ceRNA network

**Figure 4:**
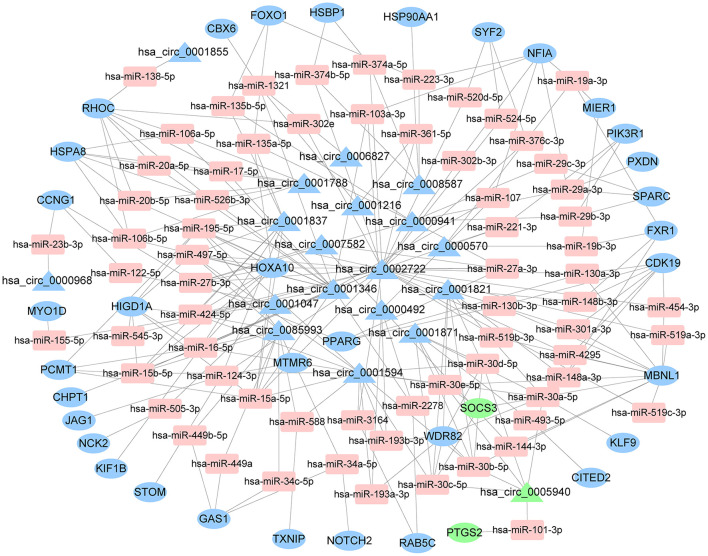
The ceRNA network of circRNA-miRNA-mRNA. The round represents mRNAs, square shapes represent miRNAs, triangles represent circRNAs, and connection represents interaction. ceRNA, competing endogenous RNA; circRNAs, circular RNAs; miRNAs, microRNAs; mRNAs, messenger RNAs.

The starBase predicted and identified 311 miRNA targets of the DEcircRNAs from the GSE144431 dataset. The interactions between 311 miRNAs and 214 DEmRNAs were analyzed using the miRcode, miRDB, TargetScan, miRmap, and miRanda. The circRNA-miRNA-mRNA ceRNA network was constructed based on DEcircRNA-miRNA and DEmRNA-miRNA pairs, including 68 miRNAs, 19 circRNAs, and 36 mRNAs (Figure 4).

### PPI network construction and hub genes identification

**Figure 5:**
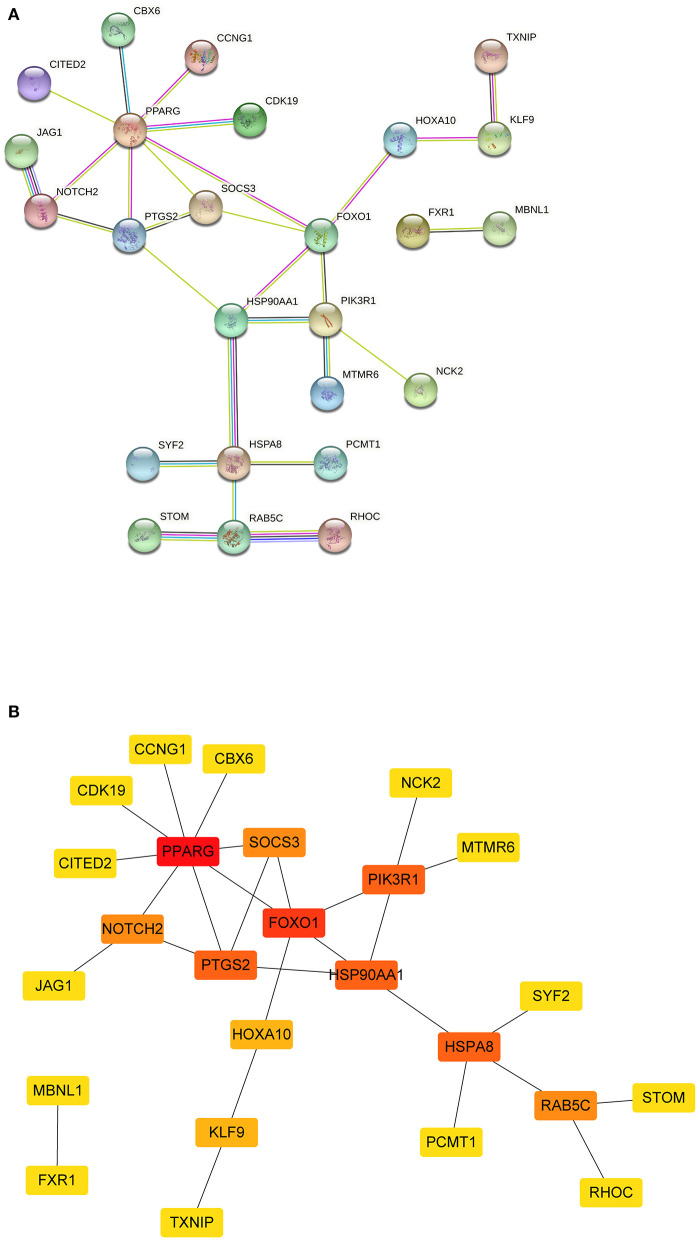
The PPI network of mRNAs from ceRNA network. **(A)** The results of the STRING database showed the protein-protein interaction network. Yellow line, interactions form textmining; blue-green line, known interactions from curated databases; dark blue, predicted interactions form gene co-occurrence; black line, interactions form co-expression; purple line, known interactions from experimentally determined; light blue, interactions form protein homology. **(B)** Square shapes represent genes, and lines represent interaction relationships. PPI, protein-protein interaction; mRNAs, messenger RNAs; ceRNA, competing endogenous RNA.

The PPI network was constructed using the STRING database based on the mRNAs from the ceRNA network. After excluding the genes that failed to interact, PPI network consisted of 25 nodes and 27 edges (Figure 5). Subsequently, nine hub genes were identified; among the genes that were upregulated were *SOCS3* (suppressor of cytokine signaling 3) and *PTGS2* (prostaglandin-endoperoxide synthase 2). The downregulated genes were *NOTCH2* (notch receptor 2), *RAB5C* (*RAB5C*, member RAS oncogene family), *HSP90AA1* (heat shock protein 90 alpha family class A member 1), *HSPA8* [heat shock protein family A (Hsp70) member 8] and *PIK3R1* (phosphoinositide-3-kinase regulatory subunit 1), *PPARG* (peroxisome proliferator-activated receptor gamma), and *FOXO1* (forkhead box O1). Among them, two key genes were identified: *PPARG* and *FOXO1*.

### Composition of the infiltrating immune cells in AAA

**Figure 6:**
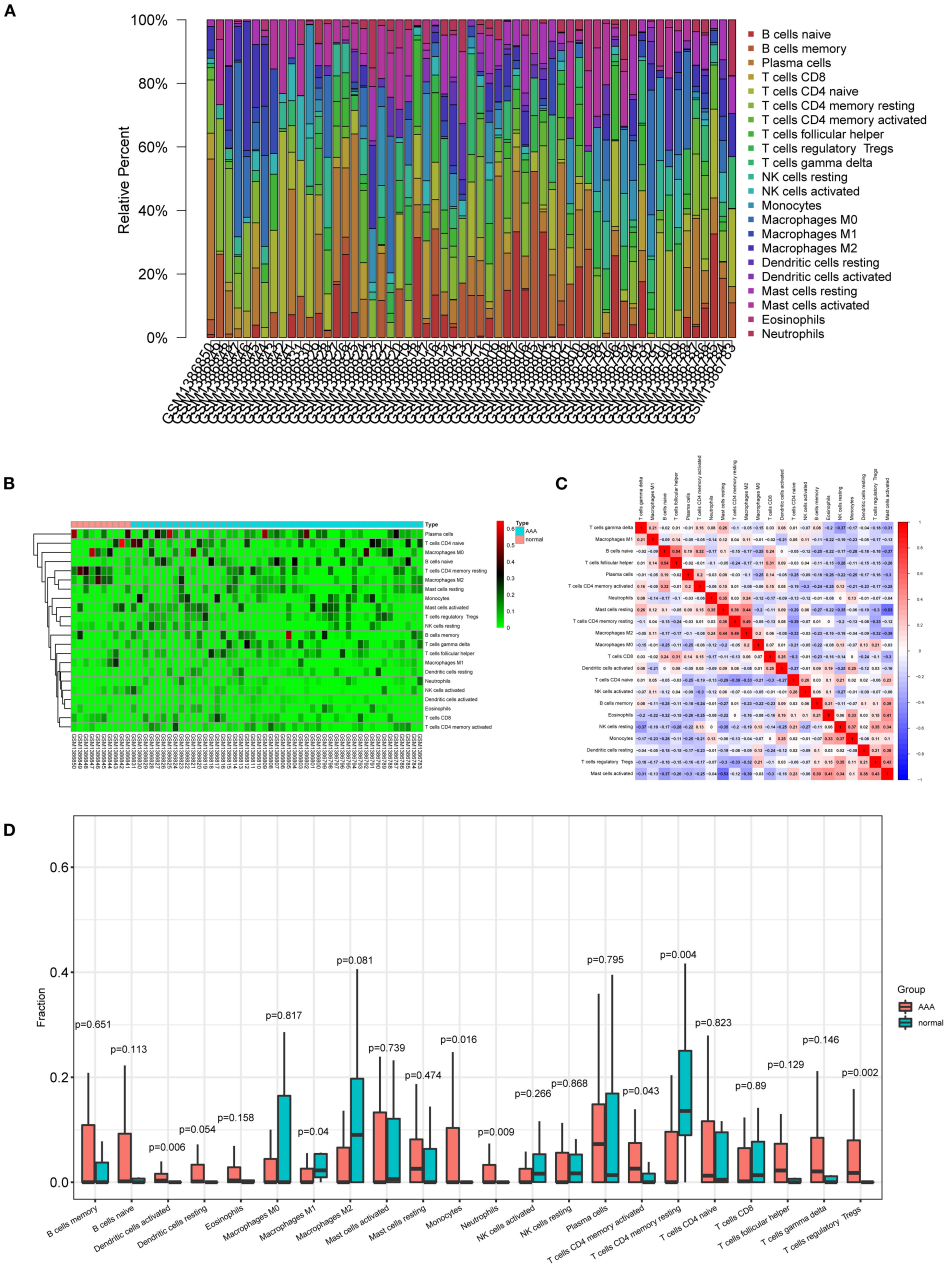
Composition of infiltrating immune cells in aortic tissues. **(A)** Distribution of immune cell types in each sample. **(B)** Heat map of infiltrating immune cells. **(C)** The correlation among infiltrating immune cells. **(D)** Violin plot of infiltrating immune cells.

The proportions and composition of 22 infiltrating immune cells were compared between the aortic tissues of AAA patients and healthy control samples by CIBERSORT algorithm (Figures 6A,B). The relationships between the abundance of 22 immune cells cells are shown in Figure 6C. The correlation analysis revealed a positive correlation between follicular helper T cells and naive B cells (*R* = 0.54) and a negative correlation between activated mast cells and resting mast cells (*R* = −0.53). Other immune cell subpopulations were weak to moderately correlated. Other immune cell subpopulations were weak to moderately correlated.

The relative proportions of infiltrating immune cells between AAA patients and healthy control samples were analyzed by Wilcoxon rank-sum test. Compared with the controls, activated dendritic cells (*p* = 0.006), monocytes (*p* = 0.016), neutrophils (*p* = 0.009), activated CD4 memory T cells (*p* = 0.043), and regulatory T cells (*p* = 0.002) were significantly enriched in AAA patients (Figure 6D; Supplementary Figure S6). The M1 macrophages (*p* = 0.04) and resting CD4 memory T cells (*p* = 0.004) were significantly less enriched in AAA samples compared with the controls (Figure 6D; Supplementary Figure S6).

### Co-expression patterns of infiltrating immune cells and key genes

**Figure 7:**
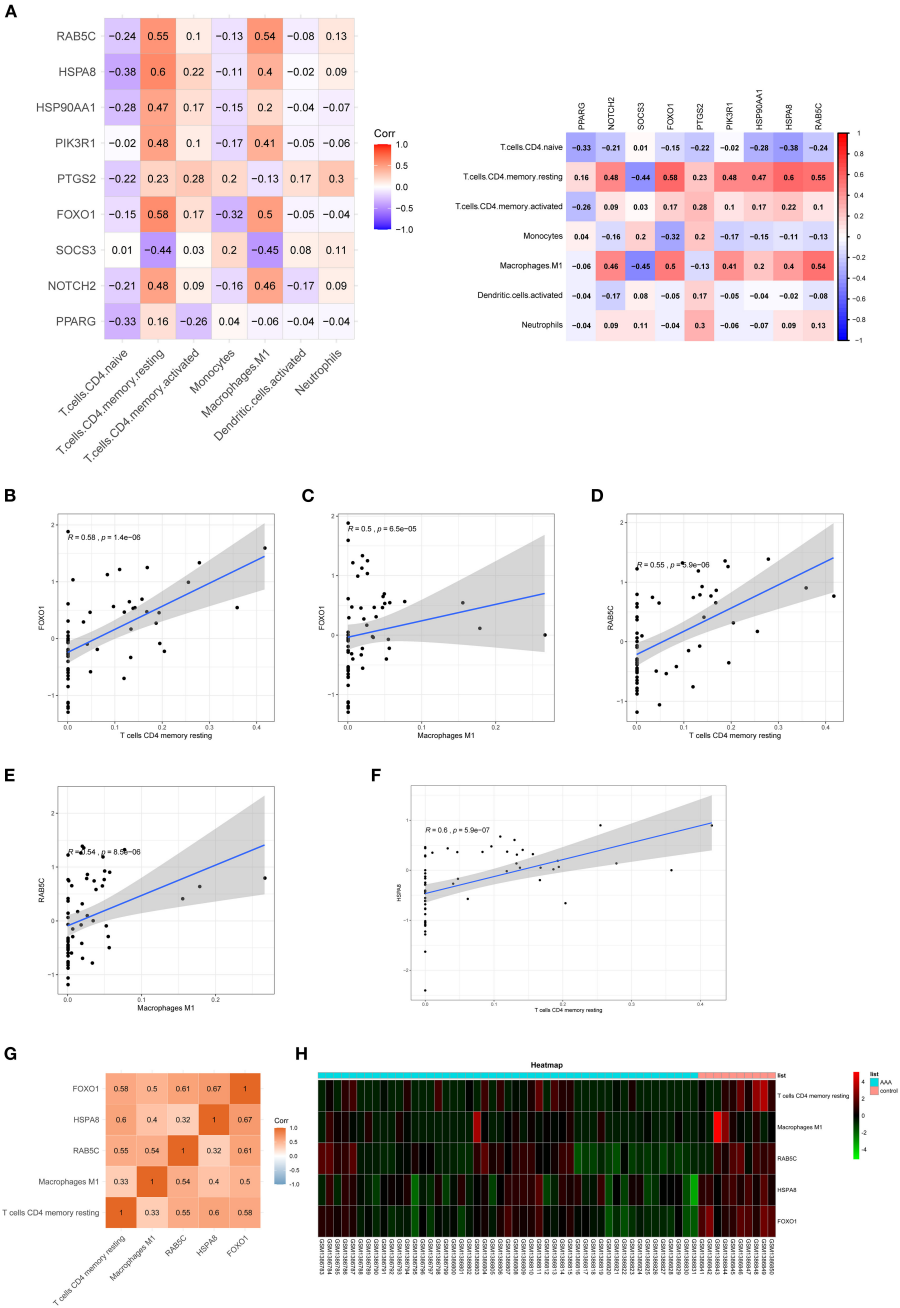
Co-expression patterns of infiltrating immune cells and key genes. **(A)** The correlation between hub genes of ceRNA network and differentially expressed infiltrating immune cells. **(B–F)** The significantly correlated pairs with correlation coefficient > 0.5 and *p* < 0.001. **(G)** The correlation between key genes (*FOXO1, RAB5C*, and *HSPA8*) and differentially expressed infiltrating immune cells. **(H)** Heat map of key genes (*FOXO1, RAB5C*, and *HSPA8*) and differentially expressed infiltrating immune cells. mRNAs, messenger RNAs; ceRNA, competing endogenous RNA.

The correlation between hub genes of the ceRNA network and differentially expressed infiltrating immune cells was estimated using Spearman's rank correlation analysis (Figures 7A–F). The *RAB5C* gene was positively correlated with resting CD4 memory T cells and M1 macrophages (*R* = 0.55 and *R* = 0.54, respectively). *HSPA8* gene was positively correlated with resting CD4 memory T cells (*R* = 0.60). The *FOXO1* gene was positively correlated with resting CD4 memory T cells and M1 macrophages (*R* = 0.58 and *R* = 0.50, respectively). The correlation between hub genes (*FOXO1, RAB5C*, and *HSPA8*) and M1 macrophages was estimated using Spearman's rank correlation analysis (Figure 7G). The expression levels of M1 macrophages and hub genes are illustrated using the heatmap (Figure 7H).

### Clinical specimen validation

**Figure 8:**
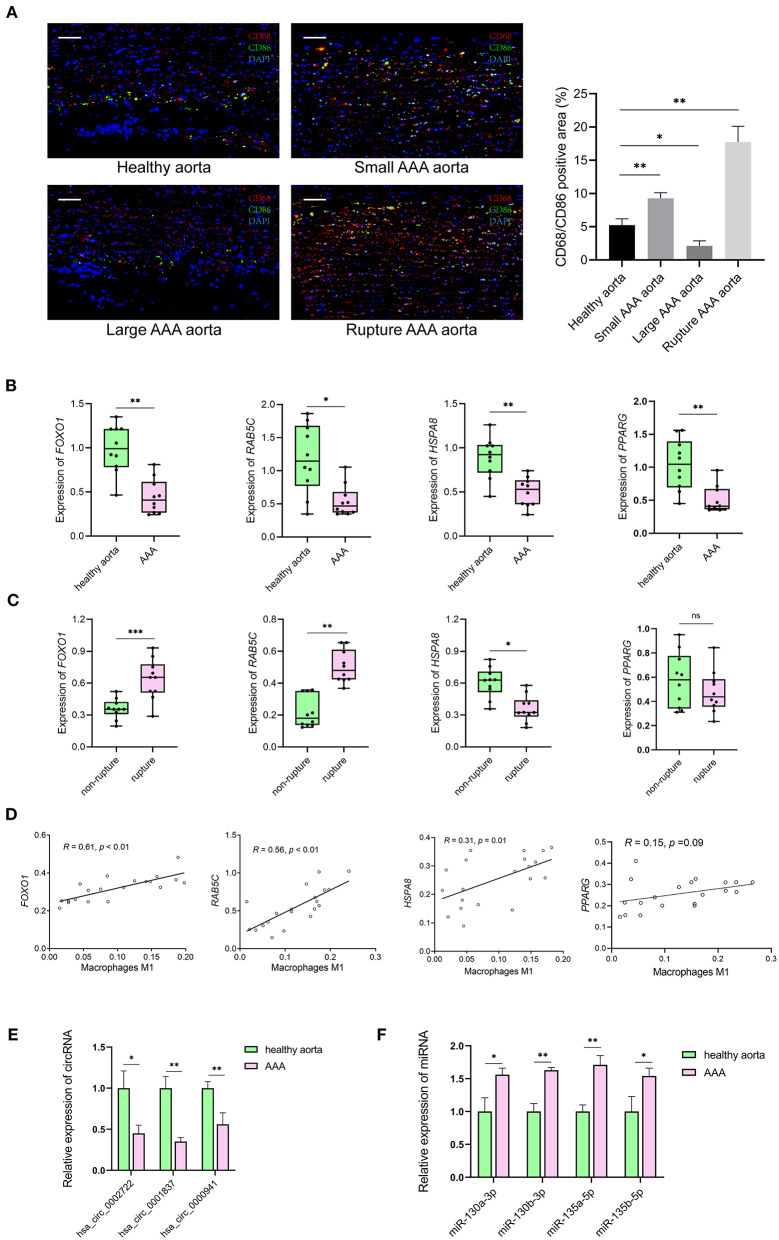
The results of preliminary clinical specimen validation. **(A)** Immunofluorescence staining of CD68 (red), CD86 (green) and DAPI (blue) in tissue samples of patients with AAA and controls. Scale bar = 100 μm. ^*^p < 0.05, ^**^p < 0.01, Student's *t*-test. **(B)** The expression levels of *RAB5C, HSPA8, FOXO1, PPARG* genes in tissue samples of AAA vs. controls. *n* = 10 in each group, ^*^p < 0.05, ^**^p < 0.01, Student's *t*-test. **(C)** The expression levels of *RAB5C, HSPA8, FOXO1, PPARG* genes in tissue samples of non-rupture AAA vs. rupture AAA. *n* = 10 in each group, ^*^p < 0.05, Student's *t*-test. **(D)** Correlation curves between the levels of M1 macrophages and key genes in tissue samples of patients with AAA and controls. Total *n* = 20. **(E)** Quantification of the relative expression levels of hsa_circ_0002722, hsa_circ_0001837, hsa_circ_0000941 in tissue samples of AAA vs. controls. *n* = 3 in each group, ^*^p < 0.05, ^**^p < 0.01, Student's *t*-test. **(F)** Quantification of the relative expression levels of miR-130a-3p, miR-130b-3p, miR-135a-5p, miR-135b-5p in tissue samples of AAA vs. controls. *n* = 3 in each group, ^*^p < 0.05, ^**^p < 0.01, Student's *t*-test.

Resting CD4 memory T cells are important cell types of the immune system and play a crucial role in resistance to pathogens. They are the reservoir for human immunodeficiency virus-1, rendering unrealistic hopes of virus eradication with current antiretroviral regimens (24). Few studies show the involvement of resting CD4 memory T cells in biological processes related to AAA. M1 macrophages circulate in the peripheral blood and reside in specific tissues, thereby conferring protection by attacking foreign substances such as bacteria and engulfing them by phagocytosis. We validated the expression of the M1 macrophage marker in clinical specimens, i.e., abdominal aortas, from AAA patients and healthy control samples. The immunofluorescence staining of CD68 and CD86 showed the infiltrating M1 macrophages were downregulated in large AAA samples (abdominal aorta diameter >55 mm), upregulated in both small AAA samples (abdominal aorta diameter ≤ 55 mm) and ruptured AAA samples compared to the healthy aorta (Figure 8A).

The mRNA levels of *FOXO1, RAB5C, HSPA8*, and *PPARG* were significantly decreased in AAA than in healthy control samples (Figure 8B). Significantly lower levels of *HSPA8* and higher levels of *FOXO1* and *RAB5C* in ruptured AAA than in unruptured AAA samples were observed (Figure 8C). The correlation between hub genes (*FOXO1, RAB5C, HSPA8*, and *PPARG*) and M1 macrophages in the clinical specimens was estimated using Spearman's rank correlation analysis (Figure 8D). The M1 macrophages positively correlated with *FOXO1* and *RAB5C* (*R* = 0.61 and *R* = 0.56, respectively). In addition, RNA expression of *FOXO1* and *PPARG* in clinical specimens were detected using RT-qPCR, confirming that hsa_circ_0002722-miR-130a/b-3p-*PPARG* and hsa_circ_0001837/hsa_circ_0000941-miR-135a/b-5p-*FOXO1* were associated with the pathogenesis of AAA (Figures 8E,F).

## Discussion

AAA is a chronic vascular disease with potentially fatal outcomes (25). Microarray-based screening methods helped identify new diagnostic and therapeutic targets. Evidence suggests the role of circRNAs in the pathogenesis of AAA (4). Unlike traditional linear RNA, circRNA has a unique covalent closed circular structure, which confers high stability and tolerance to exonuclease activity (26). According to the ceRNA hypothesis, circRNAs act as miRNA sponges, which affects and inhibits miRNA activity and alters the expression of miRNA target genes (27). To understand and explore the effect of circRNA on gene expression *via* miRNA, in this study, the ceRNA network was constructed, and immune infiltration in AAA was analyzed using bioinformatics tools.

We integrated mRNAs with the same regulated trend in the NCBI_GEO database (GSE47472 and GSE57691) and obtained 214 DEmRNAs. GO analysis revealed that these DEmRNAs were significantly enriched in the circulatory system development and negative regulation of cell population proliferation. These processes play a key role in the crucial lesion formation in vascular diseases like AAA. Consistent with the results from Batra et al., KEGG analysis revealed that the oxidative phosphorylation and TNF signaling pathway are closely related to AAA pathogenesis (28, 29). After screening out the DEcircRNAs in the NCBI_GEO database (GSE144431), we predicted the DEcircRNA-miRNA and miRNA-DEmRNA pairs that construct the ceRNA network. Hub genes play a central role in the biological mechanisms underlying the potential pathogenesis of AAAs. Two key genes, namely, *PPARG* and *FOXO1*, among hub genes in the PPI network were identified.

*PPARG* are transcription factors of the nuclear hormone receptor family that binds to peroxisome proliferators. On activation by a ligand, the nuclear receptor binds to DNA-specific PPARG response elements and regulates the transcription of its target genes (30). Studies in the Ang II-induced AAA mouse model reveal that *PPARG* upregulates the expression of anti-inflammatory cytokines such as IL-10, thereby slowing the process of AAA development and rupture (31). The activators of *PPARG*, such as rosiglitazone and pioglitazone, have been shown to alleviate the development and rupture of Ang II-induced aneurysms in mouse models (32–34). Besides, this study also found that *PPARG* may exert its impacts through hsa_circ_0002722-miR-130a/b-3p. The downregulation of *PPARG* in SMC increases pro- inflammatory factors such as MMP and OPN, thereby promoting SMC proliferation, migration, and vascular remodeling, which is associated with atherosclerosis (35, 36). Additionally, *PPARG* can also act *via* hsa_circ_0002722-miR-130a/b-3p. miR-130a promotes inflammation which accelerates disease progression in atherosclerosis by downregulating the expression of *PPARG* (37). Similarly, previous studies show upregulation of miR-130b, specifically in AAA tissue (38). Further reports suggest polymorphisms in PPARG are associated with the development and progression of AAA (39). Therefore, the changes in *PPARG* expression can alter the inflammatory response and SMC function, making it a potential therapeutic target for AAA.

*FOXO1* is a transcription factor that acts as a master switch to regulate the apoptosis of multiple cell types, including cardiomyocytes, pulmonary artery SMC, and endothelial cells (40). *FOXO1*-mediated SMC apoptosis has been reported to regulate plaque instability in advanced atherosclerotic lesions, providing new ideas for AAA formation, as SMC apoptosis is a classic pathological feature of AAA (41). *FOXO1* is also involved in cell migration, invasion, proliferation, and physiological processes such as inflammation and autophagy (42, 43). It has been shown to promote the migration, invasion, and inflammatory response of human umbilical vein endothelial cells, leading to the development and progression of a cerebral aneurysm (44). Hou et al. discovered that *FOXO1* promoted the proliferation of SMC, which contributes to the progression of cerebral aneurysm and atherosclerosis (45, 46). Moreover, *FOXO1*-mediated cellular autophagy plays a vital role in a variety of diseases, such as liver steatosis, cancer, cerebral ischemia/reperfusion injury, diabetic kidney disease, and oxidative damage (47–51). Reports suggest metformin acts as an inhibitor of autophagy and reduces the Ang II-induced AAA in mouse models, suggesting that *FOXO1* may regulate AAA pathogenesis (52). Further, results from this study reveal, *FOXO1* may exert its impacts through hsa_circ_0001837/hsa_circ_0000941-miR-135a/b-5p. It has been shown that the expression of miR-135a/b was upregulated in AAAs, and the mechanism by which they function in AAA remains to be investigated (38). A recent study suggests that miR-135a-5p inhibits SMC proliferation and migration by inactivating *FOXO1*, which is consistent with our finding (53). Although *FOXO1* has been extensively studied in cardiovascular diseases, the role of *FOXO1* in AAA requires further investigation.

AAA is caused by multiple factors, such as immune cell infiltration, inflammation, and extracellular matrix remodeling (54). This study identified the proportion of infiltrating immune cells in aortic tissues with differentially expressed immune cells, including M1 macrophages. As M1 macrophage polarization is key in promoting AAA formation, it has been widely accepted to play a part in AAA pathogenesis (4). In the Ang II-induced AAA mouse model, the Chemokine C-C motif ligand contributes to the AAA development and pathogenesis by promoting the M1 polarization of macrophages (55). Furthermore, it has been noted that miR-144-5p is a novel regulator of AAA pathology, inhibiting the M1 polarization of macrophages (56). Our results show the downregulation of M1 macrophages in AAA patients samples compared with healthy control samples. The majority of AAAs in GSE57691 were large AAAs, showing downregulation of M1 macrophages compared to controls. To validate the expression of M1 macrophages in different stages of AAA, we used clinical samples for validation. Compared to the healthy aorta, the expression of the M1 macrophages was downregulated in large-diameter AAA aorta samples. However, it was upregulated in small diameter and ruptured AAA aorta samples. This may be because AAA is a dynamic vascular disease in which M1 macrophages are initially recruited to the injured aorta early in the pathogenesis to induce inflammation. The infiltration of M1 macrophages in AAAs is associated with the long-term effects of multiple factors (4). As the AAA progresses, M1 macrophages cause chronic inflammation, which prevents the repair of the injured aorta. In large aorta AAAs, the M2 polarization of macrophages reduces inflammation, contributing to wound healing, followed by the downregulation of M1 macrophages (57, 58). Our result demonstrates that the M1 polarization of macrophages may change over time in AAA formation, and the massive infiltration of M1 macrophages may be associated with AAA rupture.

To explore the potential regulatory mechanisms of genes in infiltration immune cells, we performed the correlation analysis between hub genes and differentially expressed immune cells in aortic tissues of patients with AAA. It has been shown that hub genes, such as *FOXO1, RAB5C*, and *HSPA8*, were positively correlated with M1 macrophages or resting CD4 memory T cells. The expression of *FOXO1, RAB5C*, and *HSPA8* in aortic samples was downregulated in AAA compared to the healthy control samples Furthermore, *HSPA8* levels were low, and FOXO1 and RAB5C were higher in ruptured AAA samples compared to unruptured AAA samples. Macrophage polarization plays an important role in various diseases, such as atherosclerosis, tissue inflammation, and abdominal aortic aneurysm. However, the role of resting CD4 memory T cells in AAA has not yet been reported. A previous study shows the involvement of *FOXO1* in macrophage polarization; hence the overexpression of *FOXO1* drives macrophages to M1 phenotypes (59). *FOXO1*-mediated M1 polarization has been demonstrated in gastric cancer, periodontal bone loss, and NAFLD and can be investigated as potential pathogenesis of AAA (60, 61). It is tempting to postulate that the correlation analysis provides new insights into the mechanisms of the immune system of AAA. Further in-depth investigation is required to establish concrete conclusions.

Some limitations include that the study was conducted on a small sample size, and the data were obtained from only three microarray datasets. However, it is important to note the challenges of obtaining AAA samples. Only 22 immune cells were included, which fails to incorporate the complexities of the immune microenvironment and requires further investigation. Finally, the underlying regulatory mechanisms of the ceRNA network and their relationship with immune cell infiltration were not elucidated, and other functional experiments are required.

In summary, *PPARG, FOXO1, RAB5C*, and *HSPA8* likely play significant roles in AAA. Besides, M1 macrophages and resting CD4 memory T cells could be involved in AAA formation. A ceRNA network was established, hsa_circ_0002722-miR-130a/b-3p-*PPARG* and hsa_circ_0001837/hsa_circ_0000941-miR-135a/b-5p-*FOXO1*, may be associated with the pathogenesis of AAA. Although the expression of *FOXO1, RAB5C, HSPA8, PPARG*, and M1 macrophages were verified using clinical specimens, further studies are required to establish a correlation between DEmRNAs and differentially expressed infiltrating immune cells. This study enhances our understanding of the biological role of ceRNA and infiltrating immune cells in the pathogenesis of AAA. This may provide potential therapeutic insights for AAA, which requires further validation.

## Data availability statement

The datasets presented in this study can be found in online repositories. The names of the repository/repositories and accession number(s) can be found in the article/Supplementary material.

## Ethics statement

The studies involving human participants were reviewed and approved by Institutional Review Board of the Shanghai Jiaotong University School of Medicine, Renji Hospital. The patients/participants provided their written informed consent to participate in this study.

## Author contributions

LC and SY draft the article and contribute to the conception, design, acquisition, and interpretation of data. LC, SW, ZW, YL, and YX contribute to acquisition and analysis of data. SY and GX revise the manuscript critically for important intellectual content and make final approval of the version to be published. All authors contributed to the article and approved the submitted version.

## Funding

This work was supported by the National Natural Science Foundation of China (Grants No. 81873526).

## Conflict of interest

The authors declare that the research was conducted in the absence of any commercial or financial relationships that could be construed as a potential conflict of interest.

## Publisher's note

All claims expressed in this article are solely those of the authors and do not necessarily represent those of their affiliated organizations, or those of the publisher, the editors and the reviewers. Any product that may be evaluated in this article, or claim that may be made by its manufacturer, is not guaranteed or endorsed by the publisher.
