# Supplementary material for: Construction and analysis of competing endogenous RNA network and patterns of immune infiltration in abdominal aortic aneurysm
Source: Front Cardiovasc Med. 2022 Aug 4;9:955838. doi: 10.3389/fcvm.2022.955838 (PMC9386163; doi:10.3389/fcvm.2022.955838)
Supplement: Supplementary file 1 [file Data_Sheet_1.docx]

## Supplementary Table 1. Primers for PCR (Human)

| **Primer** | **Forward Primer (5’ to 3’)** | **Reverse Primer (5’ to 3’)** |
| --- | --- | --- |
| *GAPDH* | GGAGCGAGATCCCTCCAAAAT | GGCTGTTGTCATACTTCTCATGG |
| *FOXO1* | TCGTCATAATCTGTCCCTACACA | CGGCTTCGGCTCTTAGCAAA |
| *RAB5C* | CCGCTTTGTCAAGGGACAGTT | AGGCTGTGATACCGCTCCT |
| *HSPA8* | ACCTACTCTTGTGTGGGTGTT | GACATAGCTTGGAGTGGTTCG |
| *PPARG* | GGGATCAGCTCCGTGGATCT | TGCACTTTGGTACTCTTGAAGTT |
| U6 | ATTGGAACGATACAGAGAAGATT | GGAACGCTTCACGAATTTG |
| miR-130a-3p | AACACGCCAGTGCAATGTTAA | GTCGTATCCAGTGCAGGGT |
| miR-130b-3p | CAGUGCAAUGAUGAAAGGGCAU | GCCCUUUCAUCAUUGCACUGUU |
| miR-135a-5p | AACCCTGCTCGCAGTATTTGAG | GCGGCAGTATGGCTTTTTATTCC |
| miR-135b-5p | AGCTATGGCTTTTCATTCCTATG | CTCAACTGGTGTCGTGGAGTC |
| hsa_circ_0002722 | GCTGCCCATGCCAAATAGTTAC | AGGGTAGAAAAGGAGAGCTTCAA |
| hsa_circ_0001837 | GGACACGGACACGGATCTTC | GATGTCATTTTGGAGCCCCG |
| hsa_circ_0000941 | AAGAGAATTGGAGCTGCGGG | AAATCTCCGCAGGATGCTCT |

**Supplementary Table 2. Top20 gene Ontology term enrichment analysis for differentially expressed mRNAs**

| **Term** | **Count** | **p_value** | **Genes** |
| --- | --- | --- | --- |
| GO:0043231-intracellular membrane-bounded organelle | 154 | 9.97E-07 | ACLY, AK4, ALOX15B, ARL17A, ARL5A, ARSB, ATF4, ATP6V1C1, ATP6V1D, AZIN1, BAG3, BBX, BOLA2, C19orf12, CAV2, CBX6, CCNG1, CCNL1, CDK19, CHMP5, CHPT1, CITED2, CLN5, CNIH1, CNOT8, COA3, COL13A1, COMMD5, COX5B, CRTAP, CSNK2A2, CYFIP2, DBI, DDX19A, DIMT1, DNM1L, DUSP3, EEF1D, FANCE, FBXO11, FBXO32, FDFT1, FGFRL1, FOXO1, FOXO3, FXR1, GGNBP2, GPRC5C, GTF2A2, HES6, HIGD1A, HIPK2, HNRNPU, HOXA10, HSBP1, HSP90AA1, HSPA8, INSIG2, KDELR1, KIF1B, KLF9, LARGE1, LGR6, LSM2, LSM3, MAP3K7CL, MBNL1, METTL22, MGMT, MIER1, MPC2, MRPL33, MRPS12, MRPS21, MTMR6, MTX2, MYO1D, NAA20, NACC2, NCK2, NDN, NDUFS3, NFIA, NFIC, NHP2, NONO, NOTCH2, NR2F6, NUPR1, OCRL, OLFM1, OSBPL5, P3H3, PACSIN2, PADI4, PARVA, PDGFA, PFKM, PHF2, PIK3R1, PLN, PNPO, POLR1E, POLR2H, PPARG, PREPL, PRKDC, PRKRA, PSMG1, PTGS2, PXDN, PXMP2, QARS, R3HCC1, RAB2B, RAB5C, RCN1, RHOC, RPA2, RPS27, RRAGA, SCAMP1, SDHA, SF3B6, SKP1, SNRPB2, SNRPF, SNU13, SORBS1, SPARC, STAU1, STOM, STX2, SYF2, TBXA2R, TC2N, TIMM22, TMED2, TMEM14A, TMEM98, TMX4, TPRKB, TRAPPC2L, TRAPPC4, TSEN15, TXNIP, UBE4B, UBL5, UGP2, UQCRB, UQCRQ, UXT, WDR82, ZNF462 |
| GO:0005634-nucleus | 101 | 0.005369 | ACLY, ALOX15B, ATF4, AZIN1, BAG3, BBX, BOLA2, CAV2, CBX6, CCNG1, CCNL1, CDK19, CHMP5, CITED2, CNOT8, COMMD5, CSNK2A2, CYFIP2, DDX19A, DIMT1, DUSP3, EEF1D, FANCE, FBXO11, FBXO32, FOXO1, FOXO3, FXR1, GGNBP2, GTF2A2, HES6, HIGD1A, HIPK2, HNRNPU, HOXA10, HSBP1, HSP90AA1, HSPA8, KLF9, LSM2, LSM3, MAP3K7CL, MBNL1, METTL22, MGMT, MIER1, MPC2, MTMR6, MTX2, NAA20, NACC2, NDN, NDUFS3, NFIA, NFIC, NHP2, NONO, NOTCH2, NR2F6, NUPR1, OCRL, PACSIN2, PADI4, PARVA, PFKM, PHF2, PIK3R1, PNPO, POLR1E, POLR2H, PPARG, PREPL, PRKDC, PRKRA, PSMG1, R3HCC1, RHOC, RPA2, RPS27, RRAGA, SDHA, SF3B6, SKP1, SNRPB2, SNRPF, SNU13, SORBS1, SPARC, SYF2, TBXA2R, TC2N, TMX4, TPRKB, TSEN15, TXNIP, UBE4B, UBL5, UGP2, UXT, WDR82, ZNF462 |
| GO:0005829-cytosol | 80 | 0.000255 | ACLY, AKR1C2, ALOX15B, ASB8, ASNS, ATP6V1C1, ATP6V1D, AZIN1, BAG3, BBX, BOLA2, C19orf12, CDK19, CHMP5, CLN5, CNOT8, COMMD5, CSNK2A2, CYFIP2, DAAM1, DIMT1, DNM1L, DUSP3, EEF1D, FBXO11, FBXO32, FOXO1, FOXO3, FXR1, G3BP2, HSBP1, HSP90AA1, HSPA8, IL2RB, KLF9, LSM2, LSM3, MAP3K7CL, MBNL1, MTMR6, MYLK, MYO1D, NAA20, NCK2, NDN, OCRL, OSBPL5, PACSIN2, PADI4, PARVA, PCMT1, PDE4B, PDE5A, PFKM, PHKB, PIK3R1, PNPO, POLR2H, PPARG, PPFIA1, PREPL, PRKDC, PRKRA, PSMG1, QARS, RHOC, RPS27, RRAGA, SKP1, SNRPF, SOCS3, SORBS1, SPECC1L, STAU1, TPM1, TPRKB, TRAPPC2L, TRAPPC4, TXNIP, UGP2 |
| GO:0007275-multicellular organism development | 74 | 0.001898 | AK4, ALOX15B, ARSB, ASNS, ATF4, BAG3, BBX, CAV2, CITED2, CLDN5, CLN5, COL13A1, CSNK2A2, CXCL8, CYFIP2, DNM1L, EDNRA, EFNA1, EFNB2, FEZ2, FGFRL1, FOXO1, FXR1, GGNBP2, HES6, HIPK2, HNRNPU, HOXA10, HSBP1, HSP90AA1, INSIG2, JAG1, KDELR1, LARGE1, LGR6, MBNL1, MYLK, MYO1D, NCK2, NDN, NDUFS3, NFIA, NFIC, NOTCH2, NR2F6, NUPR1, OCRL, OLFM1, PARVA, PDGFA, PHF2, PIK3R1, PLN, PMP22, PPARG, PRKDC, PRKRA, PSMG1, PTGS2, QARS, SDHA, SF3B6, SOCS3, SPARC, STX2, SYF2, TAGLN, TMED2, TMEM98, TPM1, TRAPPC4, TXNIP, UBE4B, UQCRQ |
| GO:0031981-nuclear lumen | 72 | 0.001172 | ACLY, ATF4, BBX, CBX6, CCNG1, CCNL1, CDK19, CITED2, COMMD5, CSNK2A2, DIMT1, DUSP3, EEF1D, FANCE, FBXO11, FBXO32, FOXO1, FOXO3, FXR1, GTF2A2, HES6, HIGD1A, HIPK2, HNRNPU, HOXA10, HSBP1, HSP90AA1, HSPA8, KLF9, LSM2, LSM3, MBNL1, METTL22, MGMT, MIER1, MTX2, NACC2, NDN, NDUFS3, NFIA, NFIC, NHP2, NONO, NOTCH2, NR2F6, NUPR1, PACSIN2, PADI4, PHF2, PNPO, POLR1E, POLR2H, PPARG, PRKDC, PRKRA, PSMG1, RPA2, RPS27, SDHA, SF3B6, SKP1, SNRPB2, SNRPF, SNU13, SORBS1, SPARC, SYF2, TBXA2R, TSEN15, UXT, WDR82, ZNF462 |
| GO:0005654-nucleoplasm | 65 | 0.000288 | ACLY, ATF4, BBX, CBX6, CCNG1, CCNL1, CDK19, CITED2, COMMD5, CSNK2A2, DIMT1, DUSP3, EEF1D, FANCE, FBXO11, FBXO32, FOXO1, FOXO3, GTF2A2, HIGD1A, HIPK2, HNRNPU, HSBP1, HSP90AA1, HSPA8, KLF9, LSM2, LSM3, MBNL1, METTL22, MGMT, MIER1, NACC2, NDN, NDUFS3, NFIA, NFIC, NHP2, NONO, NOTCH2, NR2F6, NUPR1, PACSIN2, PADI4, PHF2, PNPO, POLR1E, POLR2H, PPARG, PRKDC, PRKRA, PSMG1, RPA2, RPS27, SF3B6, SKP1, SNRPB2, SNRPF, SNU13, SYF2, TBXA2R, TSEN15, UXT, WDR82, ZNF462 |
| GO:0048513-animal organ development | 56 | 0.000114 | AK4, ALOX15B, ASNS, BAG3, BBX, CAV2, CITED2, CLDN5, CLN5, COL13A1, CSNK2A2, CXCL8, EDNRA, EFNA1, EFNB2, FGFRL1, FOXO1, FXR1, GGNBP2, HIPK2, HNRNPU, HOXA10, INSIG2, JAG1, KDELR1, LARGE1, MYLK, MYO1D, NDUFS3, NFIA, NFIC, NOTCH2, NUPR1, OLFM1, PARVA, PDGFA, PHF2, PIK3R1, PLN, PPARG, PRKDC, PRKRA, PSMG1, PTGS2, QARS, SOCS3, SPARC, STX2, SYF2, TAGLN, TMED2, TMEM98, TPM1, TXNIP, UBE4B, UQCRQ |
| GO:0034641-cellular nitrogen compound metabolic process | 55 | 0.001704 | ACLY, AK4, ASNS, ATF4, AZIN1, CCNL1, CLN5, CNOT8, COX5B, DBI, DIMT1, EEF1D, FANCE, FOXO3, GTF2A2, HNRNPU, HSP90AA1, HSPA8, LSM2, LSM3, MBNL1, MGMT, MPC2, MRPL33, MRPS12, MRPS21, NDN, NFIC, NHP2, NONO, NOTCH2, NR2F6, PDE4B, PDE5A, PFKM, PNPO, POLR1E, POLR2H, PPARG, PRKDC, PRKRA, PTGS2, QARS, RNASE4, RPA2, RPS27, SF3B6, SNRPB2, SNRPF, SNU13, SYF2, TPRKB, TSEN15, UBL5, UGP2 |
| GO:0031090-organelle membrane | 55 | 0.003497 | ATP6V1C1, ATP6V1D, C19orf12, CAV2, CD58, CHMP5, CHPT1, CLN5, CNIH1, COA3, COX5B, DDX19A, DNM1L, FDFT1, FOXO3, GPRC5C, HIGD1A, HSPA8, INSIG2, KDELR1, KIF1B, LARGE1, LGR6, MPC2, MRPL33, MRPS12, MRPS21, MTX2, NCK2, NDUFS3, NOTCH2, OCRL, OSBPL5, PACSIN2, PDGFA, PLN, PTGS2, PXMP2, RAB2B, RAB5C, RRAGA, SCAMP1, SDHA, SPARC, STOM, SYPL1, TIMM22, TMED2, TMEM14A, TMEM98, TMX4, TRAPPC2L, TRAPPC4, UQCRB, UQCRQ |
| GO:0031982-vesicle | 55 | 0.021662 | ACLY, AIF1L, ALOX15B, ARSB, ATP1A2, ATP6V1C1, ATP6V1D, C5orf46, CAV2, CD58, CHMP5, CLN5, CNIH1, CPNE8, CSNK2A2, CYFIP2, DBI, DNM1L, FAM234A, FGFRL1, GGNBP2, GPRC5C, GSTCD, HSP90AA1, HSPA8, IL2RB, KDELR1, KIF1B, LGR6, MYO1D, NCK2, OCRL, PACSIN2, PCMT1, PDE4B, PDGFA, PLN, PLPP1, PXDN, RAB2B, RAB5C, RARRES1, RHOC, RRAGA, SCAMP1, SPARC, STAU1, STOM, STX2, SYPL1, TBXA2R, TMED2, TMEM98, TRAPPC4, UGP2 |
| GO:0006725-cellular aromatic compound metabolic process | 50 | 0.001362 | ACLY, AK4, AKR1C2, ATF4, CCNL1, CNOT8, COX5B, DBI, DIMT1, FANCE, FOXO3, GTF2A2, HNRNPU, HSP90AA1, HSPA8, LSM2, LSM3, MBNL1, MGMT, MPC2, NDN, NFIC, NHP2, NONO, NOTCH2, NR2F6, PDE4B, PDE5A, PFKM, PNPO, POLR1E, POLR2H, PPARG, PRKDC, PRKRA, PTGS2, QARS, RNASE4, RPA2, RPS27, SF3B6, SNRPB2, SNRPF, SNU13, SYF2, TPRKB, TRPC1, TSEN15, UBL5, UGP2 |
| GO:0022607-cellular component assembly | 48 | 5.55E-05 | AIF1L, ATP6V1D, BAG3, BOLA2, CAV2, CHMP5, CLDN5, CNIH1, COA3, CYFIP2, DNM1L, EFNB2, FGFRL1, G3BP2, GTF2A2, HSP90AA1, IL2RB, LSM3, MYLK, NACC2, NCK2, NDUFS3, NUPR1, OCRL, PACSIN2, PADI4, PARVA, PDGFA, PFKM, PLN, PMP22, POLR1E, PSMG1, PXDN, RHOC, RPA2, RPS27, SKP1, SNRPF, SNU13, SORBS1, STOM, STX2, TMED2, TPM1, TRAPPC2L, TRAPPC4, UQCRB |
| GO:0046483-heterocycle metabolic process | 48 | 0.003021 | ACLY, AK4, ATF4, CCNL1, CNOT8, COX5B, DBI, DIMT1, FANCE, FOXO3, GTF2A2, HNRNPU, HSP90AA1, HSPA8, LSM2, LSM3, MBNL1, MGMT, MPC2, NDN, NFIC, NHP2, NONO, NOTCH2, NR2F6, PDE4B, PDE5A, PFKM, PNPO, POLR1E, POLR2H, PPARG, PRKDC, PRKRA, PTGS2, QARS, RNASE4, RPA2, RPS27, SF3B6, SNRPB2, SNRPF, SNU13, SYF2, TPRKB, TSEN15, UBL5, UGP2 |
| GO:0006139-nucleobase-containing compound metabolic process | 47 | 0.001428 | ACLY, AK4, ATF4, CCNL1, CNOT8, COX5B, DBI, DIMT1, FANCE, FOXO3, GTF2A2, HNRNPU, HSP90AA1, HSPA8, LSM2, LSM3, MBNL1, MGMT, MPC2, NDN, NFIC, NHP2, NONO, NOTCH2, NR2F6, PDE4B, PDE5A, PFKM, POLR1E, POLR2H, PPARG, PRKDC, PRKRA, PTGS2, QARS, RNASE4, RPA2, RPS27, SF3B6, SNRPB2, SNRPF, SNU13, SYF2, TPRKB, TSEN15, UBL5, UGP2 |
| GO:0031324-negative regulation of cellular metabolic process | 44 | 0.001293 | ATF4, BAG3, CBX6, CITED2, CNOT8, CRTAP, CSNK2A2, DUSP3, EFNA1, FEZ2, FOXO1, FOXO3, FXR1, GAS1, GGNBP2, HES6, HIPK2, HNRNPU, HSBP1, HSPA8, INSIG2, MIER1, MYADM, MYO1D, NACC2, NCK2, NFIA, NFIC, NONO, NOTCH2, NR2F6, NUPR1, PDGFA, PPARG, PRKDC, PTGS2, QARS, RARRES1, RRAGA, SOCS3, TMEM98, TRIM44, TXNIP, UXT |
| GO:0071310-cellular response to organic substance | 43 | 0.00061 | AKR1C2, ASNS, ATF4, ATP1A2, ATP6V1C1, ATP6V1D, BAG3, BOLA2, CAV2, CD58, CDK19, CHMP5, CLDN5, CXCL8, DNM1L, DUSP3, FBXO32, FGFRL1, FOXO1, FOXO3, GAS1, HIPK2, HNRNPU, HSP90AA1, HSPA8, IL2RB, KIF1B, KLF9, NDN, NR2F6, PDE4B, PIK3R1, PLPP1, POLR2H, PPARG, PRKDC, PTGS2, RRAGA, SKP1, SOCS3, SORBS1, SPARC, TBXA2R |
| GO:0009653-anatomical structure morphogenesis | 42 | 0.000128 | CITED2, CLDN5, COL13A1, CXCL8, CYFIP2, DNM1L, EDNRA, EFNA1, EFNB2, FEZ2, FGFRL1, FOXO1, HIPK2, HOXA10, HSBP1, HSP90AA1, INSIG2, JAG1, LGR6, MBNL1, MPZL2, MYLK, NDN, NFIC, NOTCH2, OLFM1, PACSIN2, PARVA, PDGFA, PIK3R1, PMP22, PRKDC, PRKRA, PTGS2, RHOC, SF3B6, SOCS3, STX2, SYF2, TMED2, TPM1, UBE4B |
| GO:0010467-gene expression | 39 | 0.000251 | ATF4, BOLA2, CCNL1, CLN5, CNOT8, CYFIP2, DDX19A, DIMT1, EEF1D, FOXO3, GTF2A2, HNRNPU, HSPA8, LSM2, LSM3, MBNL1, MRPL33, MRPS12, MRPS21, NAA20, NFIC, NHP2, NONO, NOTCH2, NR2F6, POLR1E, POLR2H, PPARG, PRKRA, QARS, RPS27, SF3B6, SNRPB2, SNRPF, SNU13, SYF2, TPRKB, TSEN15, UBL5 |
| GO:0007399-nervous system development | 38 | 0.008204 | AK4, ARSB, ATF4, BAG3, CLN5, CSNK2A2, CYFIP2, DNM1L, EDNRA, EFNA1, EFNB2, FEZ2, HES6, HIPK2, HSP90AA1, JAG1, LGR6, MBNL1, MYO1D, NCK2, NDN, NDUFS3, NFIA, NOTCH2, NR2F6, OLFM1, PIK3R1, PMP22, PPARG, PRKDC, PSMG1, QARS, SDHA, TMED2, TMEM98, TRAPPC4, UBE4B, UQCRQ |
| GO:0006796-phosphate-containing compound metabolic process | 37 | 0.003764 | ACLY, AK4, CCNG1, CDK19, CHPT1, COX5B, CSNK2A2, DBI, DUSP3, FDFT1, FGFRL1, HIPK2, IL2RB, MPC2, MTMR6, MYLK, NDUFS3, OCRL, OSBPL5, PDE4B, PDE5A, PDGFA, PFKM, PHKB, PIK3R1, PLPP1, PNPO, PPP4R1, PRKDC, PRKRA, PTGS2, SDHA, SKP1, SOCS3, UGP2, UQCRB, UQCRQ |

**Supplementary Table 3. Top20 genome pathway enrichment analysis for differentially expressed mRNAs**

| **Term** | **Count** | **p_value** | **Genes** |
| --- | --- | --- | --- |
| hsa03040-Spliceosome | 9 | 0.000082 | HNRNPU,HSPA8,LSM2,LSM3,SF3B6,SNRPB2,SNRPF,SNU13,SYF2 |
| hsa04932-Non-alcoholic fatty liver disease (NAFLD) | 9 | 0.000174 | ATF4,COX5B,CXCL8,NDUFS3,PIK3R1,SDHA,SOCS3,UQCRB,UQCRQ |
| hsa00190-Oxidative phosphorylation | 7 | 0.002089 | ATP6V1C1,ATP6V1D,COX5B,NDUFS3,SDHA,UQCRB,UQCRQ |
| hsa04668-TNF signaling pathway | 6 | 0.003985 | ATF4,DNM1L,JAG1,PIK3R1,PTGS2,SOCS3 |
| hsa04260-Cardiac muscle contraction | 5 | 0.006016 | ATP1A2,COX5B,TPM1,UQCRB,UQCRQ |
| hsa04211-Longevity regulating pathway | 5 | 0.006946 | ATF4,FOXO1,FOXO3,PIK3R1,PPARG |
| hsa04213-Longevity regulating pathway  multiple species | 4 | 0.009658 | FOXO1,FOXO3,HSPA8,PIK3R1 |
| hsa05215-Prostate cancer | 5 | 0.009910 | ATF4,FOXO1,HSP90AA1,PDGFA,PIK3R1 |
| hsa04137-Mitophagyanimal- | 4 | 0.011367 | ATF4,CITED2,CSNK2A2,FOXO3 |
| hsa05202-Transcriptional mis-regulation in cancer | 7 | 0.012808 | CXCL8,FOXO1,HOXA10,IL2RB,NUPR1,PDGFA,PPARG |
| hsa05016-Huntington disease | 7 | 0.015434 | COX5B,NDUFS3,POLR2H,PPARG,SDHA,UQCRB,UQCRQ |
| hsa04510-Focal adhesion | 7 | 0.017973 | CAV2,ITGA10,MYLK,PARVA,PDGFA,PIK3R1,PPP1R12C |
| hsa03018-RNA degradation | 4 | 0.021869 | CNOT8,LSM2,LSM3,PFKM |
| hsa04919-Thyroid hormone signaling pathway | 5 | 0.022278 | ATP1A2,FOXO1,NOTCH2,PIK3R1,PLN |
| hsa04152-AMPK signaling pathway | 5 | 0.023004 | FOXO1,FOXO3,PFKM,PIK3R1,PPARG |
| hsa04022-cGMP-PKG signaling pathway | 6 | 0.025349 | ATF4,ATP1A2,EDNRA,MYLK,PDE5A,PLN |
| hsa05110-Vibrio cholerae infection | 3 | 0.029749 | ATP6V1C1,ATP6V1D,KDELR1 |
| hsa05165-Human papillomavirus infection | 9 | 0.034115 | ATP6V1C1,ATP6V1D,FOXO1,HES6,ITGA10,JAG1,NOTCH2,PIK3R1,PTGS2 |
| hsa04714-Thermogenesis | 7 | 0.036610 | COA3,COX5B,NDUFS3,PPARG,SDHA,UQCRB,UQCRQ |
| hsa04910-Insulin signaling pathway | 5 | 0.037746 | FOXO1,PHKB,PIK3R1,SOCS3,SORBS1 |

**Supplementary Figure 1. GO analysis of 214 differentially expressed mRNA**

**(A-D)** The GO interaction network of 214 DEmRNAs from GSE57691 and GSE47472 (the emapplot of coexpression genes from BP enriched pathway). DEmRNAs, differentially expressed messenger RNAs; GO, Gene Ontology; BP, biological process.

**Supplementary Figure 2. GO analysis of 214 differentially expressed mRNA**

**(A-D)** The GO interaction network of 214 DEmRNAs from GSE57691 and GSE47472 (the emapplot of coexpression genes from CC enriched pathway). DEmRNAs, differentially expressed messenger RNAs; GO, Gene Ontology; CC, cellular component.

**Supplementary Figure 3. GO analysis of 214 differentially expressed mRNA**

**(A-D)** The GO interaction network of 214 DEmRNAs from GSE57691 and GSE47472 (the emapplot of coexpression genes from MF enriched pathway). DEmRNAs, differentially expressed messenger RNAs; GO, Gene Ontology; MF, molecular function.

**Supplementary Figure 4. GO analysis of 214 differentially expressed mRNA**

**(A)** Circleplot of 214 DEmRNAs by GO analysis. **(B)** Bar of 214 DEmRNAs by GO analysis. **(C)** Bubble of 214 DEmRNAs by GO analysis. **(D)** Chord plot of 214 DEmRNAs by GO analysis. DEmRNAs, differentially expressed messenger RNAs; GO, Gene Ontology.

**Supplementary Figure 5. KEGG analysis of 214 differentially expressed mRNA**

**(A-D)** The emapplot of KEGG pathways shown the “pathway–pathway” network (GSE57691 and GSE47472). DEmRNAs, differentially expressed messenger RNAs; KEGG, Kyoto Encyclopedia of Genes and Genomes.

**Supplementary Figure 6. Composition of infiltrating immune cells in aortic tissues.**

**(A)** PCA analysis of infiltrating immune cells in aortic tissues from GSE57691. **(B-H)** Differentially expressed infiltrating immune cells in aortic tissues of patients with AAA and controls.
