# Supplementary figures and images for: Construction and analysis of competing endogenous RNA network and patterns of immune infiltration in abdominal aortic aneurysm
Source: Front Cardiovasc Med. 2022 Aug 4;9:955838. doi: 10.3389/fcvm.2022.955838 (PMC9386163; doi:10.3389/fcvm.2022.955838)

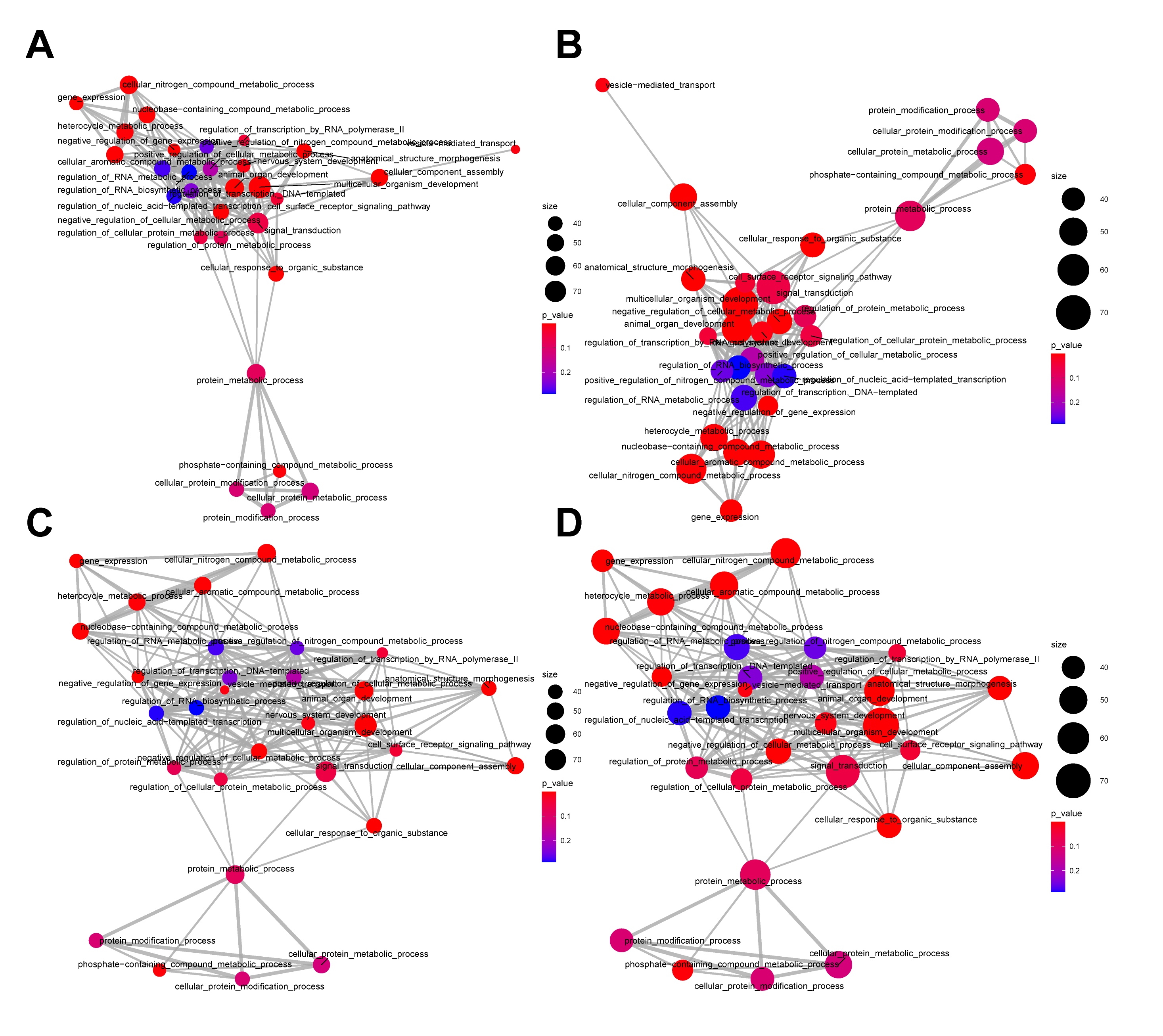

Supplement: Supplementary file 2 [file Image_1.TIF]

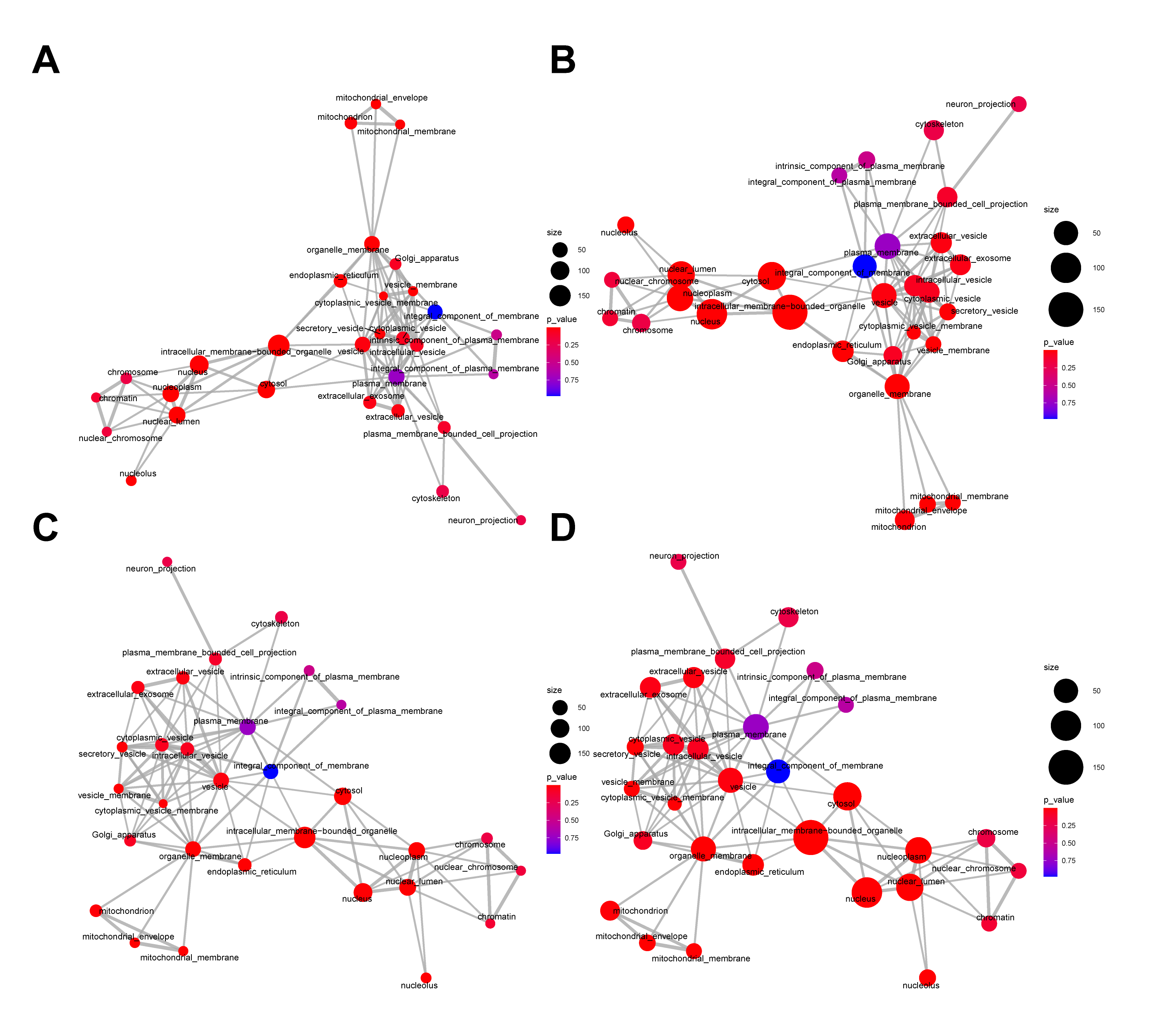

Supplement: Supplementary file 3 [file Image_2.TIF]

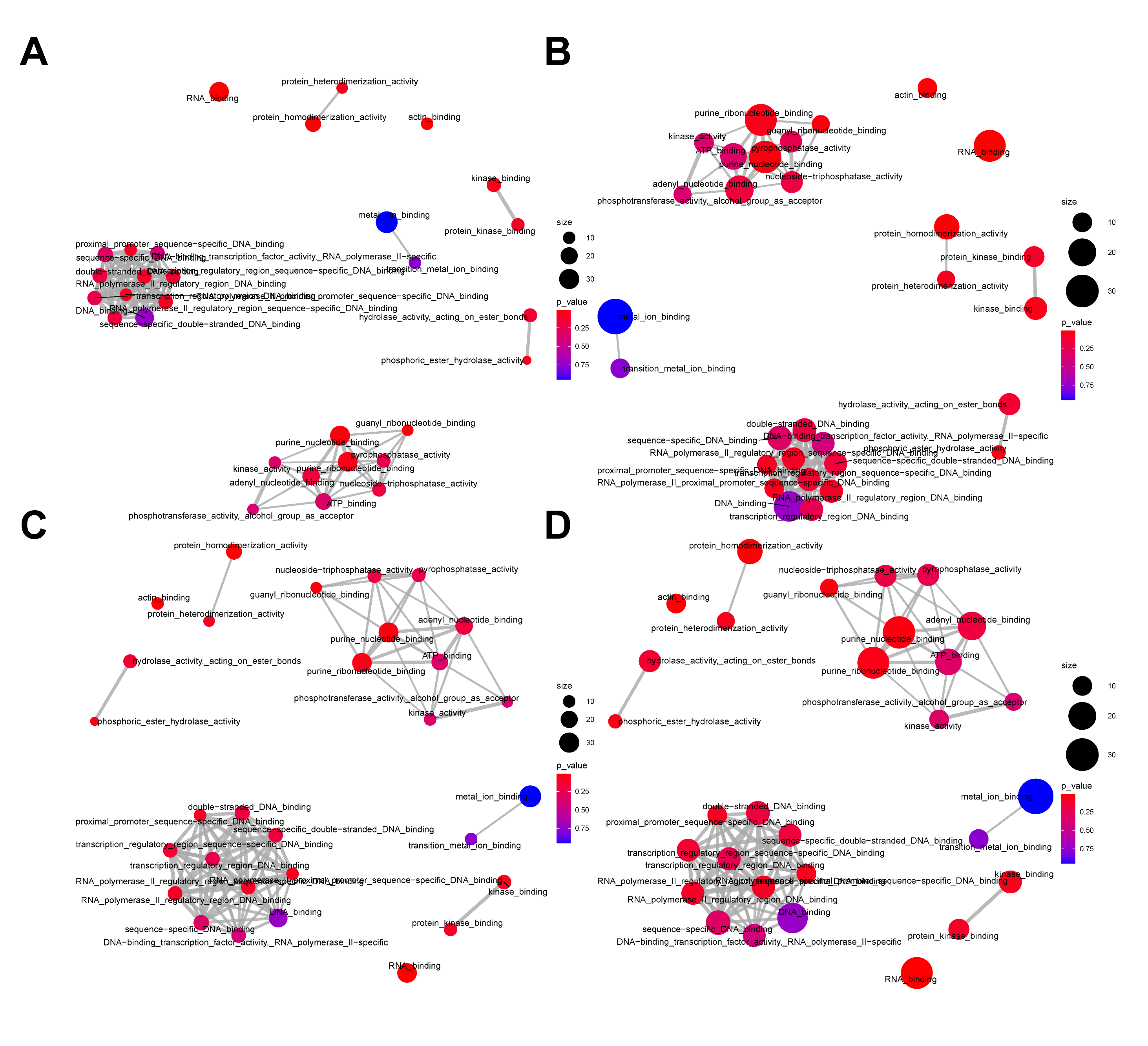

Supplement: Supplementary file 4 [file Image_3.TIF]

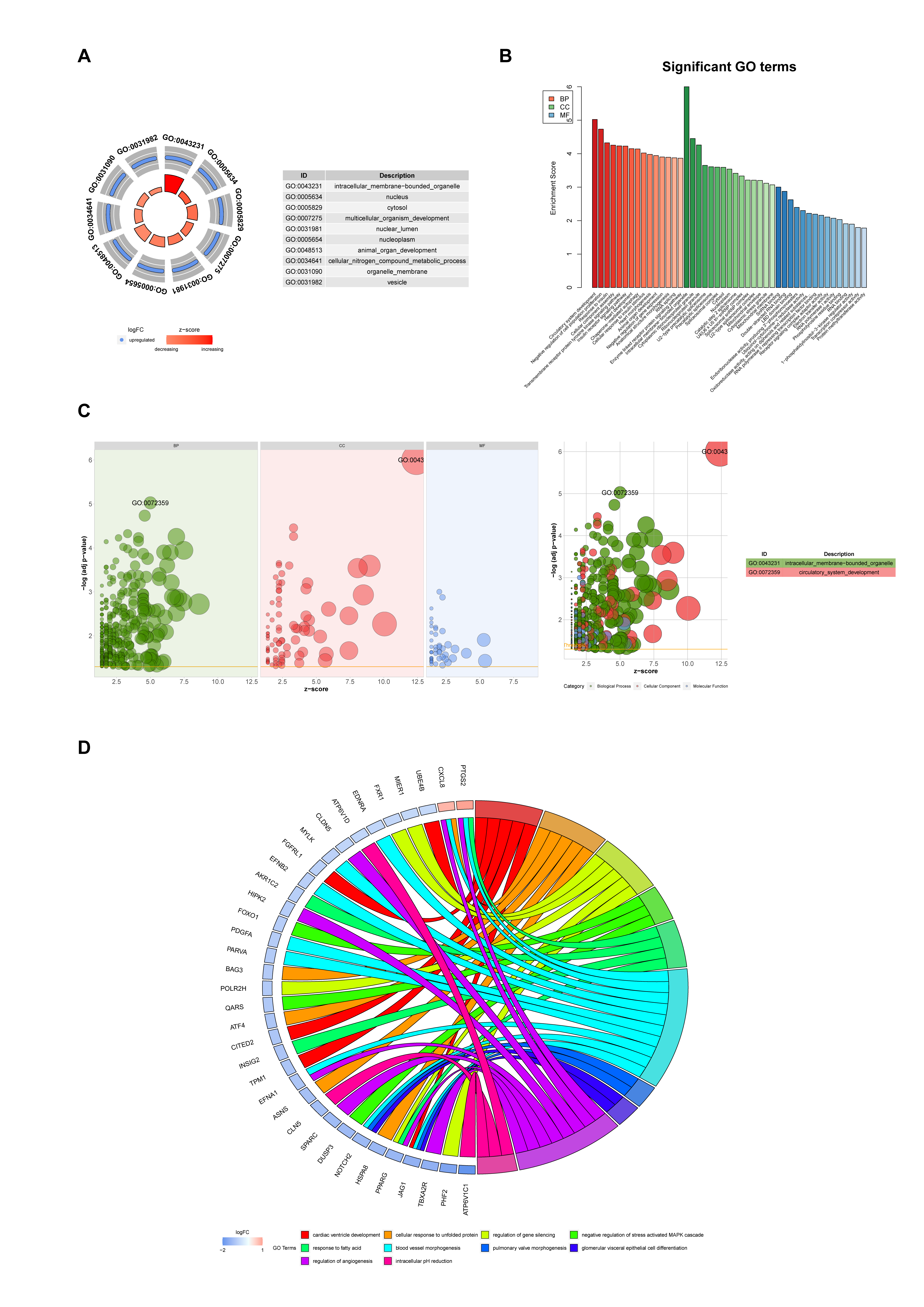

Supplement: Supplementary file 5 [file Image_4.TIF]

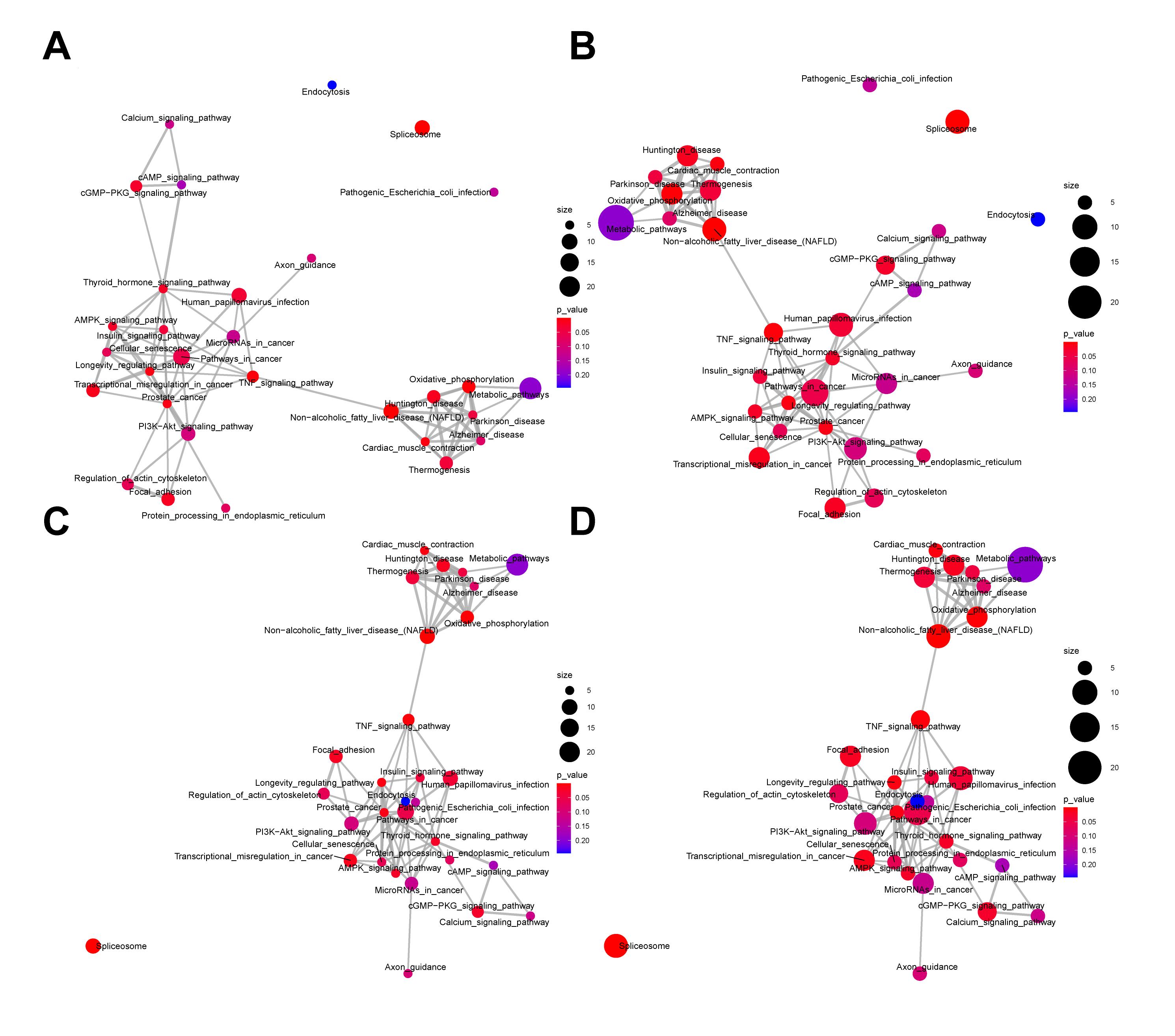

Supplement: Supplementary file 6 [file Image_5.TIF]

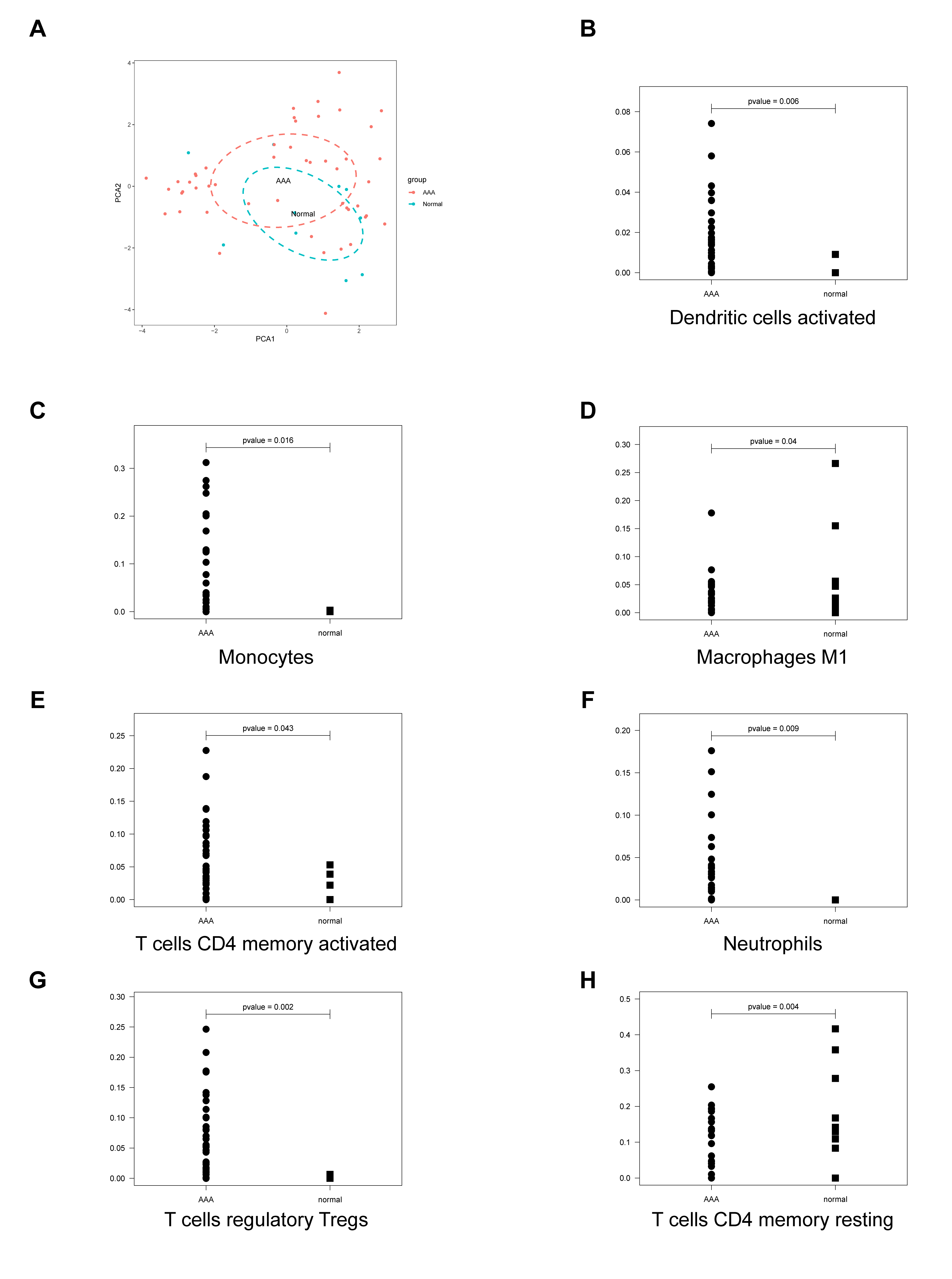

Supplement: Supplementary file 7 [file Image_6.TIF]
